# A comparative analysis of whole genome sequencing of esophageal adenocarcinoma pre- and post-chemotherapy

**DOI:** 10.1101/gr.214296.116

**Published:** 2017-06

**Authors:** Ayesha Noorani, Jan Bornschein, Andy G. Lynch, Maria Secrier, Achilleas Achilleos, Matthew Eldridge, Lawrence Bower, Jamie M.J. Weaver, Jason Crawte, Chin-Ann Ong, Nicholas Shannon, Shona MacRae, Nicola Grehan, Barbara Nutzinger, Maria O'Donovan, Richard Hardwick, Simon Tavaré, Rebecca C. Fitzgerald, on behalf of the Oesophageal Cancer Clinical and Molecular Stratification (OCCAMS) Consortium

**Affiliations:** 1 Medical Research Council Cancer Unit, Hutchison/Medical Research Council Research Centre, University of Cambridge, Cambridge CB2 0XZ, United Kingdom;; 2 Cancer Research UK Cambridge Institute, University of Cambridge, Cambridge CB2 0RE, United Kingdom;; 3 Department of Histopathology, Addenbrooke's Hospital, Cambridge CB2 0QQ, United Kingdom;; 4 Oesophago-Gastric Unit, Addenbrooke's Hospital, Cambridge CB2 0QQ, United Kingdom; 7 Medical Research Council Cancer Unit, Hutchison/Medical Research Council Research Centre, University of Cambridge, Cambridge CB2 0XZ, United Kingdom; 8 Department of Histopathology, Addenbrooke's Hospital, Cambridge CB2 0QQ, United Kingdom; 9 Cancer Research UK Cambridge Institute, University of Cambridge, Cambridge CB2 0RE, United Kingdom; 10 Oxford ComLab, University of Oxford, OX1 2JD, United Kingdom; 11 Department of Computer Science, University of Oxford, OX1 3QD, United Kingdom; 12 Cambridge University Hospitals NHS Foundation Trust, Cambridge, CB2 0QQ, United Kingdom; 13 Centre for Cancer Research and Cell Biology, Queen's University Belfast, BT9 7AB, Northern Ireland, United Kingdom; 14 Salford Royal NHS Foundation Trust, Salford, M6 8HD, United Kingdom; 15 Faculty of Medical and Human Sciences, University of Manchester, M13 9PL, United Kingdom; 16 Wigan and Leigh NHS Foundation Trust, Wigan, Manchester, WN1 2NN, United Kingdom; 17 GI Science Centre, University of Manchester, M13 9PL, United Kingdom; 18 Royal Surrey County Hospital NHS Foundation Trust, Guildford, GU2 7XX, United Kingdom; 19 Edinburgh Royal Infirmary, Edinburgh, EH16 4SA, United Kingdom; 20 Edinburgh University, Edinburgh, EH8 9YL, United Kingdom; 21 University Hospitals Birmingham NHS Foundation Trust, Birmingham, B15 2GW, United Kingdom; 22 Institute of Cancer and Genomic Sciences, University of Birmingham, B15 2TT, United Kingdom; 23 University Hospital Southampton NHS Foundation Trust, Southampton, SO16 6YD, United Kingdom; 24 Cancer Sciences Division, University of Southampton, Southampton, SO17 1BJ, United Kingdom; 25 Gloucester Royal Hospital, Gloucester, GL1 3NN, United Kingdom; 26 St Thomas's Hospital, London, SE1 7EH, United Kingdom; 27 Karolinska Institutet, SE-171 77, Stockholm, Sweden; 28 King's College London, London, WC2R 2LS, United Kingdom; 29 Plymouth Hospitals NHS Trust, Plymouth, PL6 8DH, United Kingdom; 30 Norfolk and Norwich University Hospital NHS Foundation Trust, Norwich, NR4 7UY, United Kingdom; 31 Nottingham University Hospitals NHS Trust, Nottingham, NG7 2UH, United Kingdom; 32 University College London, London, WC1E 6BT, United Kingdom; 33 Norfolk and Waveney Cellular Pathology Network, Norwich, NR4 7UY, United Kingdom; 34 Wythenshawe Hospital, Manchester, M23 9LT, United Kingdom; 35 University Hospitals Coventry and Warwickshire NHS Trust, Coventry, CV2 2DX, United Kingdom; 36 Peterborough Hospitals NHS Trust, Peterborough City Hospital, Peterborough, PE3 9GZ, United Kingdom; 37 Queen's Medical Centre, University of Nottingham, Nottingham, NG7 2UH, United Kingdom; 38 Imperial College NHS Trust, Imperial College London, W2 1NY, United Kingdom; 39 Barking Havering and Redbridge University Hospitals NHS Trust, Queen's Hospital, Romford RM7 0AG, United Kingdom; 40 Royal Stoke University Hospital, Stoke-on-Trent, ST4 6QG, United Kingdom

## Abstract

The scientific community has avoided using tissue samples from patients that have been exposed to systemic chemotherapy to infer the genomic landscape of a given cancer. Esophageal adenocarcinoma is a heterogeneous, chemoresistant tumor for which the availability and size of pretreatment endoscopic samples are limiting. This study compares whole-genome sequencing data obtained from chemo-naive and chemo-treated samples. The quality of whole-genomic sequencing data is comparable across all samples regardless of chemotherapy status. Inclusion of samples collected post-chemotherapy increased the proportion of late-stage tumors. When comparing matched pre- and post-chemotherapy samples from 10 cases, the mutational signatures, copy number, and SNV mutational profiles reflect the expected heterogeneity in this disease. Analysis of SNVs in relation to allele-specific copy-number changes pinpoints the common ancestor to a point prior to chemotherapy. For cases in which pre- and post-chemotherapy samples do show substantial differences, the timing of the divergence is near-synchronous with endoreduplication. Comparison across a large prospective cohort (62 treatment-naive, 58 chemotherapy-treated samples) reveals no significant differences in the overall mutation rate, mutation signatures, specific recurrent point mutations, or copy-number events in respect to chemotherapy status. In conclusion, whole-genome sequencing of samples obtained following neoadjuvant chemotherapy is representative of the genomic landscape of esophageal adenocarcinoma. Excluding these samples reduces the material available for cataloging and introduces a bias toward the earlier stages of cancer.

The incidence of esophageal adenocarcinoma (EAC) has increased sixfold in the last 30 yr (Lepage et al. 2013). The majority of patients present with advanced disease, and the overall survival is <15% despite advances in multimodal therapy (Jemal et al. 2011; Masclee et al. 2014). Patients who do not have distant nodal or organ metastases are considered suitable for treatment with curative intent. This generally comprises systemic chemotherapy followed by surgical excision. Chemotherapy has been shown to improve survival to >30% for those entering a curative pathway and is now an integral part of standard care either alone or in combination with radiotherapy, although the benefits of radiotherapy are greater in esophageal squamous cell carcinoma (Medical Research Council Oesophageal Cancer Working Group 2002; Cunningham et al. 2006). Complete pathological response after neoadjuvant chemotherapy is rare and constitutes <15% of all cases, highlighting that residual cancer cells often remain after this treatment (Sjoquist et al. 2011; Orditura et al. 2014).

Chemotherapeutic agents exert their effect by directly or indirectly inducing DNA damage and cell death. In EAC, three distinct classes of drugs are mainly used in combination: an intercalating agent, a platinum-derivative, and an anti-metabolite (Allum et al. 2011). Drugs such as epirubicine intercalate directly with the DNA strand and thereby disrupt further replication in rapidly dividing cells. Platinum drugs directly modify DNA through coordinate-covalent bonds between DNA and the platinum moiety, and the gross DNA damage is repaired via the nucleotide excision repair pathway (NER) if intact. 5′-Fluorouracil and derivatives target DNA metabolism and result in DNA adducts, strand breaks, or stalled/collapsed DNA replication forks. In addition, many of these drugs result in an increase of reactive oxygen species (ROS), which can in turn induce DNA damage, including single-strand DNA breaks (Woods and Turchi 2013). Hence, one might expect to see direct effects of chemotherapeutic agents on the DNA sequence, and the extent might depend on the tumor responsiveness to treatment (Rebucci and Michiels 2013).

The mutation burden in EAC is high, with 8.0 mutations/Mb (range 1.53–34.56/Mb) per haploid genome (Alexandrov et al. 2013). The genomic landscape appears to be complex and heterogeneous with a large number of point mutations occurring at very low frequency apart from *TP53* mutations, which are present in 70%–80% cases (Dulak et al. 2013; Weaver et al. 2014). Whole-genome sequencing studies and SNP arrays are providing more detail on large-scale chromosomal rearrangements that are common with evidence of catastrophic events such as chromothripsis and breakage-bridge-fusion (BFB) occuring in around one-third of patients (Dulak et al. 2013; Nones et al. 2014).

In the current study, we performed whole-genome sequencing in highly clinically annotated samples of EAC that included chemo-naive and chemo-treated samples as part of the International Cancer Genome Consortium (ICGC). We took the opportunity to critically evaluate the impact of chemotherapy on the genomic landscape. It has recently been reported from exome data that chemotherapy imposes a bottleneck on tumor evolution (Findlay et al. 2016). We therefore first sought to establish the genetic relationship between 10 matched pre- and post-chemotherapy samples and the point at which the samples diverged. After perfoming this initial analysis, we examined the single-nucleotide variant (SNV) spectrum, mutational/trinucleotide context, and copy-number aberrations in a larger cohort of 58 chemotherapy-treated and 62 chemotherapy-naive samples.

## Results

### Whole-genome sequencing of paired samples pre- and post-chemotherapy

Whole-genome sequencing data were first analysed for 10 cases from which samples were taken pre- and post-chemotherapy. The clinical details of this cohort are shown in Supplemental Table S1. Of these 10 cases, eight had a single sample taken before and after neoadjuvant chemotherapy, and three had multiple samples taken before and after treatment.

Overall, the matched samples showed the expected range of estimated tumor cellularity, overall ploidy, mutational signature composition, SNV burden, and copy-number variation, including losses of heterozygosity (LOH), as well as focal amplifications and deletions (Supplemental Table S3). Regions of LOH, amplifications, and deletions are mostly the same pre-and post-chemotherapy (with LOH always observed on the same allele for paired samples). Paired samples range from being almost identical (patient 001: 97% of the genome in the same copy-number state, 95% of SNVs called in both samples) to very altered (patient 008: 27% of the genome in the same copy-number state, 23% of SNVs called in both samples).

For each patient, we observe copy-number features present in all tumor cells prechemotherapy that are not present post-chemotherapy, and vice versa. The key question is whether these differences are a consequence of the chemotherapy or simply a reflection of heterogeneity. In seven out of nine cases (the cellularity in one case is too low to call), we identify regions that have lost heterozygosity in the prechemotherapy samples but have retained heterozygosity in the post-chemotherapy samples. This implies that the post-chemotherapy sample cannot have evolved from the prechemotherapy sample but rather they have a shared antecedent.

**Figure 1.**
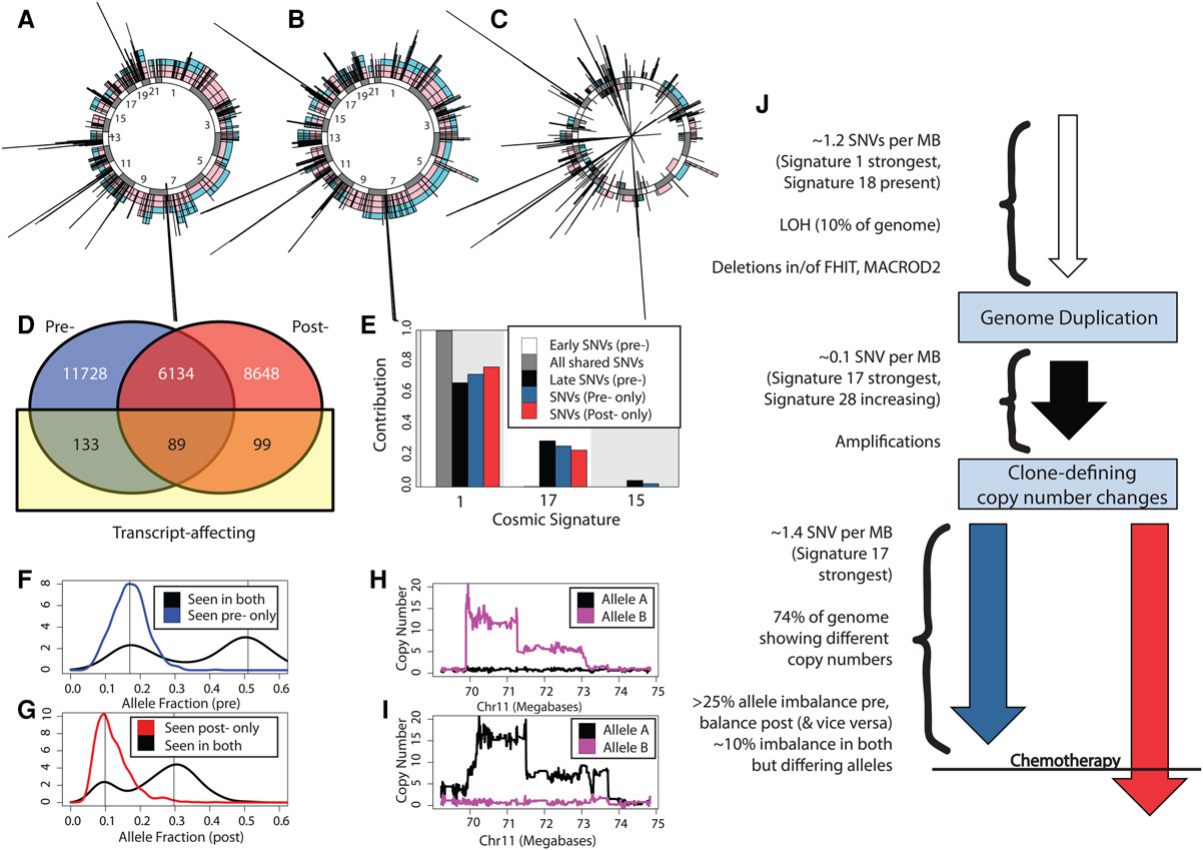
Profiling case 008 where the pre- and post-chemotherapy samples are different. (*A*–*C*) Illustrated are allele-specific copy-number states for the 22 autosomes: (*A*) prechemotherapy (alleles represented by colors), (*B*) post-chemotherapy, and (*C*) the difference between the two allele-specific copy-number profiles pre- and post-chemotherapy. Copy-number increases post- to prechemotherapy are shown outside the circle; decreases are shown inside the circle. (*D*) Venn diagram showing the numbers of SNV calls shared pre- and post-chemotherapy, classified also by whether they affect coding genes. (*E*) The mutational process signatures (reported in the Catalogue of Somatic Mutations in Cancer) that contribute substantially to the called SNVs are shown. Of the shared SNVs, approximately 6000 lie within copy-number states AA, AAB, AABB, AAA, or AAAA and can confidently be categorized as early or late (relative to their copy-number changes). The contributions for these subsets are shown also. (*F*) For regions that in the prechemotherapy sample have copy-number status AAA, we see that no SNVs unique to this sample have three copies. (*G*) For regions that in the post-chemotherapy sample have copy-number status AAA, we see that no SNVs unique to this sample that have three copies. (*H*) Illustrated are allele-specific copy numbers for a region of Chromosome 11 in the prechemotherapy sample. (*I*) Illustrated are allele-specific copy numbers for a region of Chromosome 11 in the post-chemotherapy sample. (*J*) A sketched likely timeline for this sample, although inherent to this type of data, the timings of losses are supposition.

**Figure 2.**
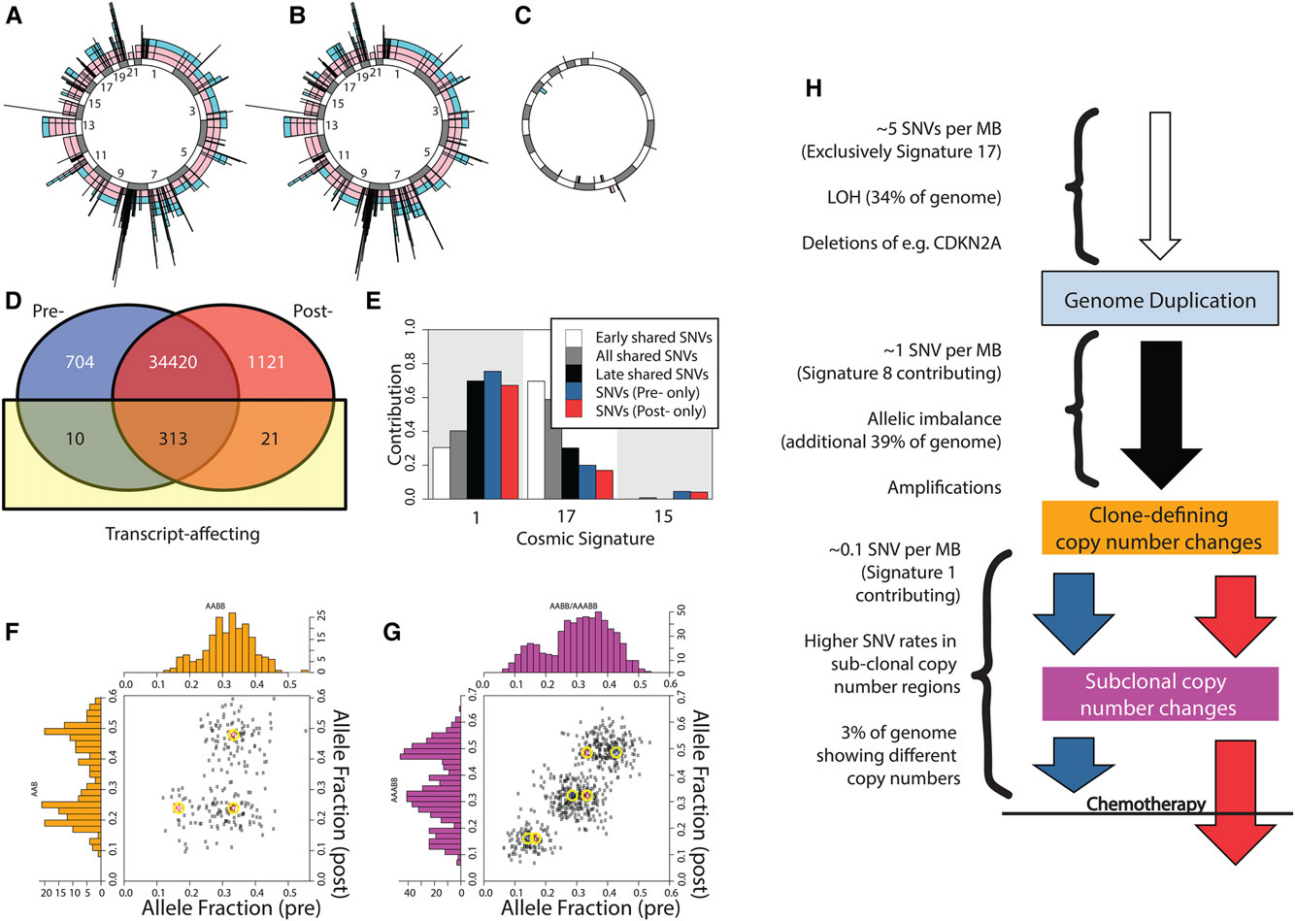
Profiling case 001 where the pre- and post-chemotherapy samples are similar. (*A*) Illustrated are allele-specific copy-number states for the 22 autosomes prechemotherapy (alleles represented by colors). (*B*) Illustrated are allele-specific copy-number states for the 22 autosomes post-chemotherapy. (*C*) Illustrating the difference between the two allele-specific copy-number profiles. Copy-number increases post- to prechemotherapy are shown outside the circle; decreases are shown inside the circle. (*D*) Venn diagram showing the numbers of SNV calls shared pre- and post-chemotherapy, classified also by whether they are transcript-affecting. (*E*) The mutational process signatures (reported at the Catalogue of Somatic Mutations in Cancer) that contribute substantially to the called SNVs are shown. Of the shared SNVs, approximately 14,000 lie within copy-number states AA, AAB, AABB, AAAA, or AAAAB and can confidently be categorized as early or late (relative to their copy-number changes). The contributions for these subsets are shown also. (*F*) Illustrating SNVs for a region that exhibits different copy-number states pre- (AABB) and post- (AAB) chemotherapy. The centers of predicted clusters for these states are indicated. Within each sample, the copy-number state appears to be consistent in 100% of tumor cells. (*G*) Illustrated are SNVs for a region that demonstrates subclonal copy-number behavior prechemotherapy. The two sets of expected cluster centers for clonal AABB (red) and AAABB (blue) solutions prechemotherapy, against AAABB post-chemotherapy, are illustrated. The lack of a fourth SNV cluster is a strong indicator that this is a subclonal loss of one copy from a previously clonal AAABB state. (*H*) A sketched likely timeline for this sample, although the timing of losses is supposition.

**Figure 3.**
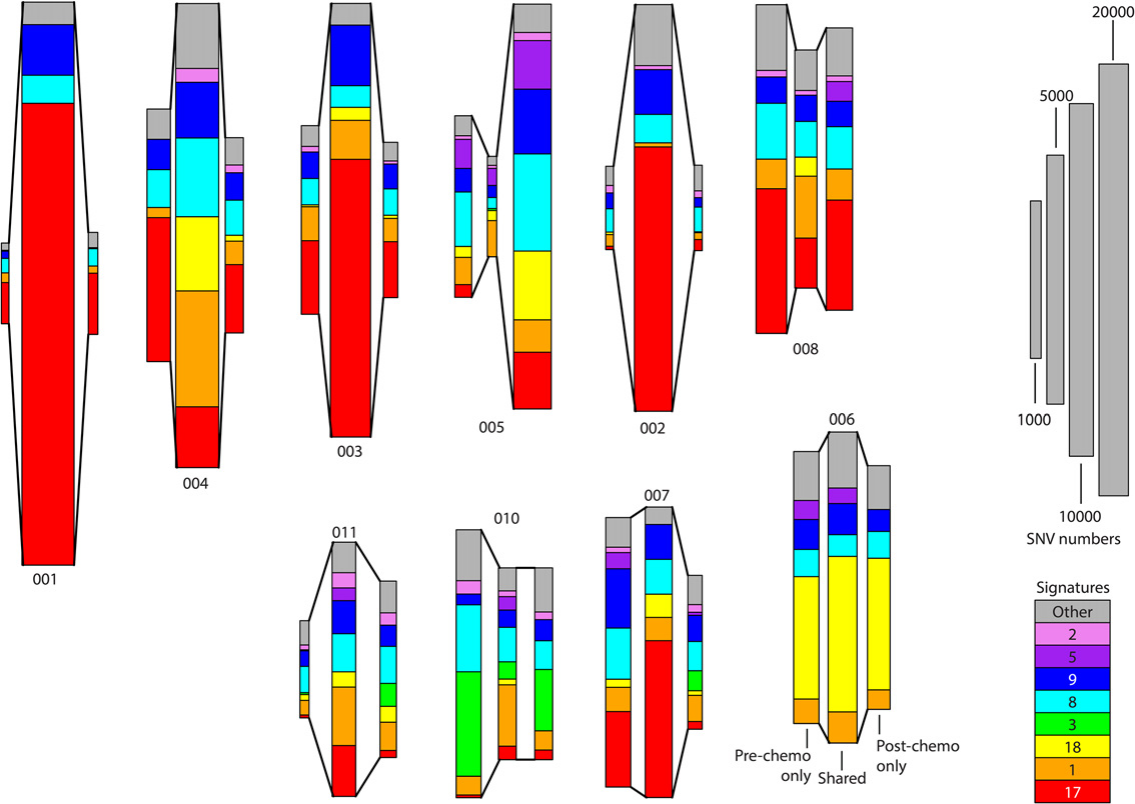
Mutational signatures of mutational context for paired samples pre- and post-neaodjuvant chemotherapy. Illustrated are the numbers of shared and unique single-nucleotide variants (SNVs) and their breakdown into 30 known signatures from COSMIC. Since inference of signatures de novo is complicated by the nonindependence and small numbers of samples, we do not attempt to do so but rather infer the breakdown using quadratic programming methods (see Methods). For each patient, three rectangles are presented showing SNVs called prechemotherapy only (*left*), shared SNV calls (*center*), and SNVs called post-chemotherapy only (*right*). The size of the rectangle indicates the number of SNVs, and the proportion of color denotes the breakdown into signatures, as indicated in the key. Only eight signatures that make sizeable contributions are individually identified.

It is informative to discuss the two extreme cases indicated above in more detail (Figs. 1, 2). In patient 008, a minority of the genome has the same copy-number state pre- and post-chemotherapy (Fig. 1A–C), and in addition, a minority of SNVs are observed both pre- and post-chemotherapy (Fig. 1D). Events that are known to be early, e.g., mutations of *TP53* and LOH of key genes (Supplemental Fig. S1), are seen to be shared, and indeed, the majority of the genome that does exhibit LOH is common to both samples (Supplemental Table S3) and always occurs on the same allele when it is common. While different mutations are observed in the pre- and post-chemotherapy samples, the same mutational processes appear to be present (Figs 1E, 3), and the AAB copy-number state is the most common (Supplemental Table S3). Unlike most of the pre- and post-samples in this cohort, the majority of focal amplifications are not shared, but convergent amplification of the *FGF* region is observed in both samples (Fig. 1H,I). One can therefore infer that clonal divergence occurred shortly after endoreduplication (Fig. 1F,G,J), and hence, the differences between the two samples are attributable to events that predate chemotherapy.

Patient 001 is a very different case, with virtually no differences pre- and post-chemotherapy (Fig. 2A–D; Supplemental Table S3). We can see some “clonal” differences between the two samples (Fig. 2F). “Subclonal” behavior prechemotherapy appears to be a recent change from a clonal state that matched the post-chemotherapy sample, indicating that although the two samples have diverged only recently (Fig. 2H; Supplemental Table S2), the differences such as they are cannot be attributed to the chemotherapy regime.

In general, when the pre- and post-chemotherapy samples show substantial differences, the timing of the divergence of the samples can be traced to being near-synchronous to endoreduplication. For the samples that show little difference, we can still trace their common ancestry to a point prior to the chemotherapy and see no evidence in the mutational signatures (Fig. 3), copy numbers (Supplemental Table S3), or key genes (Supplemental Fig. S1) to suggest that we are seeing anything other than the heterogeneity.

It is clear that the mutational signatures change over time (Figs. 1–3) and that the more recent mutations are disproportionately affected by factors affecting the power to detect SNVs (including sequencing depth, genomic complexity, and cellularity). Caution must therefore be taken in concluding that chemotherapy has had an effect on the observed mutational signatures, and we do not draw such a conclusion.

Our data suggest that differences seen pre- and post-chemotherapy are reflective of tumor heterogeneity and that either sample could be considered equally representative of the case. However, from these data on a small patient cohort, we cannot rule out the possibility of a subtle selective pressure, and in order to address this, we require larger cohorts of pre- and post-chemotherapy samples, which thus form the second part of this analysis.

### Systematic comparison of whole-genome sequencing data on a large cohort of chemotherapy-naive or chemotherapy treated samples

Our large cohort (*n* = 120), shown in Supplemental Fig. S2a, comprised 314 patients from whom there were 138 chemotherapy-naive samples taken at endoscopic diagnosis, prior to any treatment, or at the time of surgical resection if no neoadjuvant systemic chemotherapy was given, and 176 samples taken at surgery following systemic chemotherapy. For the patients receiving chemotherapy, samples were not available both before and after treatment in this cohort. A further breakdown of the samples selected for the large cohort analysis is shown in Supplemental Fig. S2b. The chemotherapy given at all centers was in line with the United Kingdom recommendation, comprising a platinum compound as a backbone generally combined with epirubicin and a 5-fluoruracil derivative. Patients receiving radiotherapy were excluded in order to maintain consistency across the cohorts. The details of the study cohort are shown in Table 1.

**Table 1.**
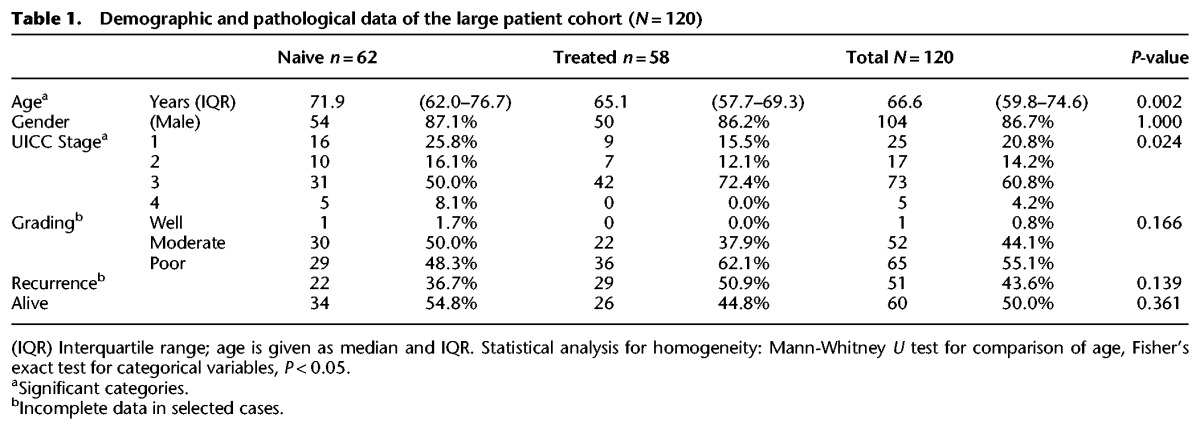
Demographic and pathological data of the large patient cohort (*N* = 120)

Patients for which chemotherapy-treated samples were sequenced were significantly younger (*P* = 0.002) and, as expected, presented at a more advanced stage of disease (*P* = 0.024) since, for those patients going down curative pathways, chemotherapy is not required for early stage tumors and patients have to be fit enough to endure toxic therapy. Thus, 25 patients (40%) with chemotherapy-naive samples went straight for surgery without neoadjuvant systemic treatment and were of an earlier stage. Histological response to neoadjuvant chemotherapy as assessed by the Mandard regression score was documented in 78 of the 95 patients (Supplemental Table S7). Of these, 16 (21%) had Mandard scores of one to three, indicating some degree of histological response. A score of four to five (present in the remaining 79%) indicates poor response to neoadjuvant treatment, as expected for this particular cancer. Although the chemotherapy-treated group showed higher recurrence rates, this was not statistically significant (*P* = 0.139). At the time of analysis, 50% of the patients were alive, with no significant difference between the treated and the chemotherapy-naive group (*P* = 0.361). Please note that these statistics reflect the earlier stage cases in the chemotherapy-naive group and are thus not reflective of the known benefit of chemotherapy shown in randomized trials for this disease.

All cases underwent a stringent pathological review of a frozen H&E section from the same sample that would be submitted for sequencing to confirm the diagnosis and ensure that the histopathological estimate of tumor cellularity exceeded 70%. Of the total cohort of *n* = 314 samples (*n* = 176 chemotherapy-treated, *n* = 138 treatment-naive), significantly more samples that were exposed to chemotherapy failed this pathology review and were therefore excluded (*n* = 98, 55.7% vs. *n* = 35, 25.4%; *P* < 0.001) (Supplemental Fig. S2b). Of the treatment-naive samples, a higher proportion of endoscopic biopsies failed the pathology review compared with surgical specimens as would be expected from their small size (*n* = 33, 30.6% vs. *n* = 2, 6.7%; *P* = 0.01).

### Genomic metrics of chemo-naive and chemo-treated samples of the large cohort

The group of treatment-naive samples contained a median of 24,449 SNVs and indels (combined), with a median absolute deviation (MAD) of 16,355, while the chemo-treated group had a median of 20,071 SNVs and indels (MAD = 12,223). The mutation rates in the chemo-naive and treated groups were similar, with a mean of 8.7 mutations/Mb for the former and 7.5 mutations/Mb for the latter (Wilcoxon rank-sum test *P*-value = 0.4).

**Figure 4.**
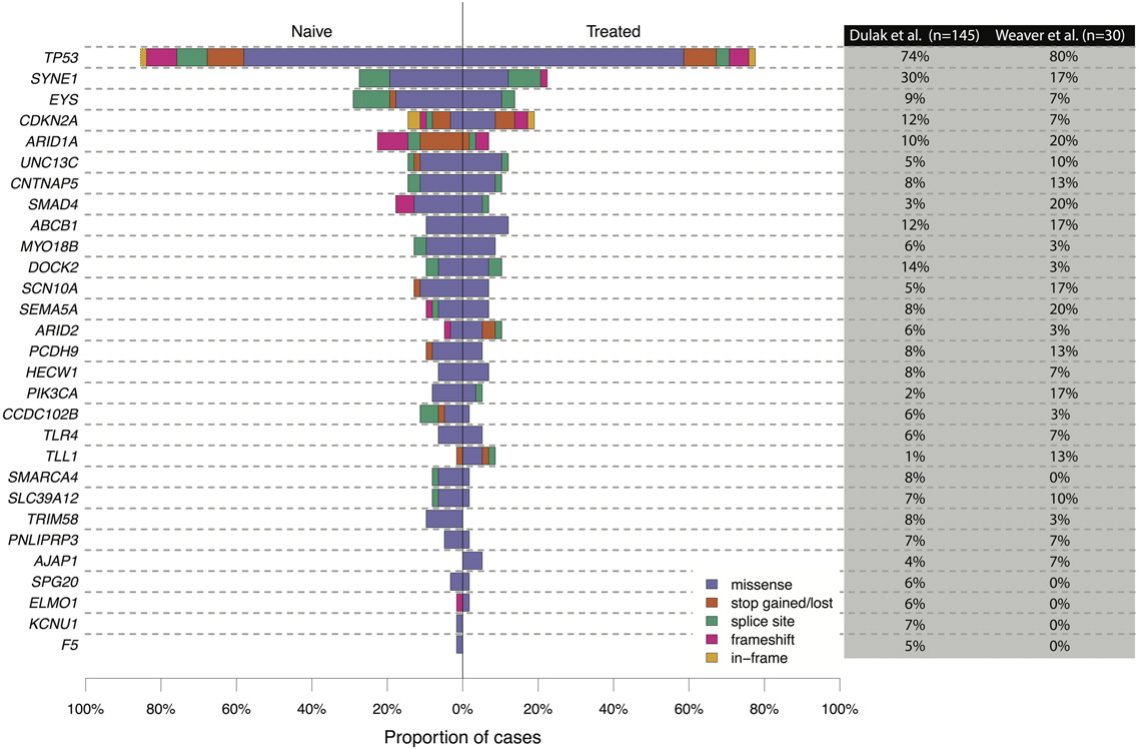
Proportion of nonsynonymous SNVs and indels in recurrently mutated genes in chemotherapy-treated and chemotherapy-naive cohorts. The genes were selected from the top-ranking genes described in either of the Dulak et al. (2013) or Weaver et al. (2014) studies. The corresponding table demonstrates the percentage of samples that had mutations in these selected genes.

Some genes were only recurrently mutated in the chemotherapy treated samples, e.g., *PTGES3L-AARSD1*, *RN7SL332P*, *AC011893.3*, *OR4D12P*, *TSPAN10*, *PPFIA3*, *C15orf39*, *SLC27A4*, and *NAA30*, in at least 15% of this group. However, the top recurrently mutated genes that have been previously characterized for EAC, which are more likely drivers in this cancer, were generally mutated in a similar proportion of cases across the two cohorts (Fig. 4; Dulak et al. 2013; Weaver et al. 2014).

**Figure 5.**
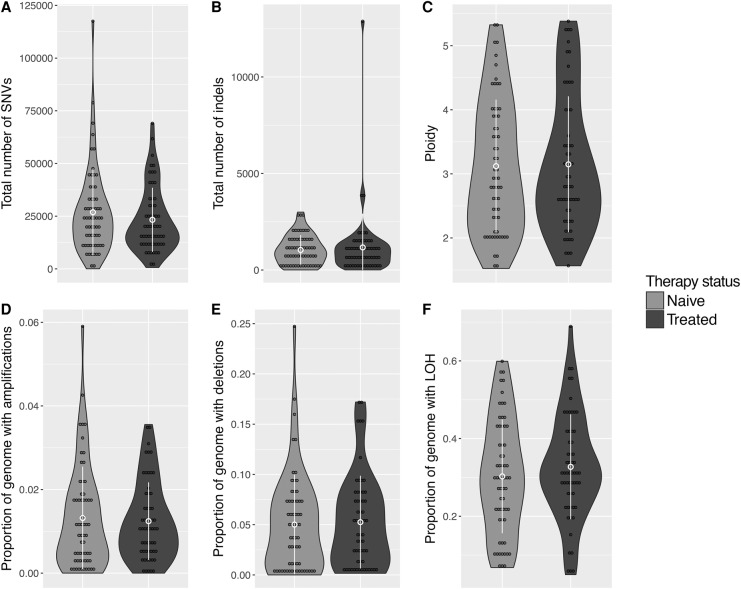
Genomic architecture in chemotherapy-naive (*n* = 62) and chemotherapy-treated (*n* = 58) samples. (*A*) Total number of SNVs, (*B*) total number of indels, (*C*) average ploidy, (*D*) percentage of the genome that is amplified (defined as copy number ≥2× the average ploidy), (*E*) percentage of the genome with deletions (defined as copy number ≤0.5× the average ploidy), and (*F*) percentage of the genome with LOH. No significant difference between naive and chemo-treated groups is observed in any case. The mean ± 1 SD are highlighted in each case.

The tissue samples in the two groups displayed similar proportions of amplifications, deletions, and LOH regions (Wilcoxon rank-sum test *P*-values >0.05) (Fig. 5; Supplemental Tables S4, S8; Supplemental Fig. S3). Furthermore, in each group, the defined genomic characteristics were similar regardless of patient age, disease stage, resection margin status (positive or negative for tumor cells), or sample source (biopsy or resection specimen), (Supplemental Table S5).

### The effect of chemotherapy on mutational spectrum analysis in the large cohort

**Figure 6.**
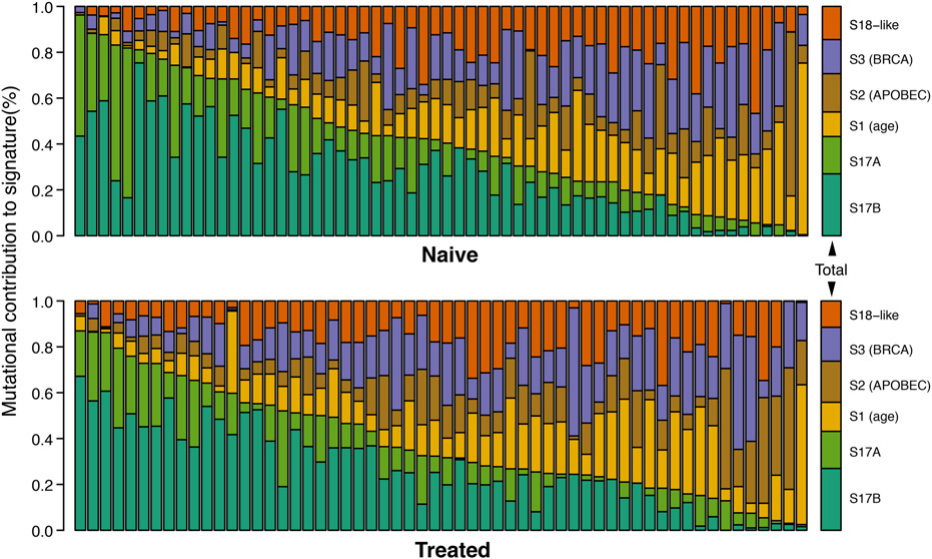
Mutational spectra analysis in chemotherapy-treated and chemotherapy-naive patient groups. The most relevant signatures for each group have been identified according to the method published by Alexandrov et al. (2013). Each bar shows the proportion of calls for the relevant signature per sample; the bars on the far *right*, the cumulative proportion for each group.

EAC mutational signatures were extracted using the method presented by Alexandrov et al. (2013). A total of six mutational signatures were identified, of which five have been previously identified in EAC and other cancer types (Dulak et al. 2013; Weaver et al. 2014). None of these five signatures have been previously associated with exposure to chemotherapy. We therefore compared the number of mutations generated by each signature within the two cohorts and did not observe any clear difference (Fig. 6). A comparison of the mutational signatures for the 10 pairs of samples pre- and post-chemotherapy again showed similar nonsignificant differences (Supplemental Fig. S6).

Since there was no significant overrepresentation of a particular trinucleotide in either group (Wilcoxon rank-sum test adjusted *P*-values >0.05) (Supplemental Fig. S4), this prompted us to repeat the analysis with a focus on C>A substitution mutations occurring at CpC dinucleotides that have been previously reported to be associated with systemic treatment with cisplatin (Meier et al. 2014). There was a significant enrichment for the cisplatin-induced mutational signature in the chemotherapy-treated cohort (Fisher's exact test *P*-value <0.0001) (Supplemental Fig. S5), in agreement with observations by Murugaesu et al. (2015).

## Discussion

In this study, we have used whole-genome sequencing, incorporating a comprehensive analysis of copy number, SNVs, and mutational signatures from prospectively collected samples with stringent pathology QC but without imposing any restriction on including samples collected from patients who had already been treated with chemotherapy.

The first aim was to ensure that inclusion of chemotherapy-exposed tissues did not result in poor quality samples in terms of low cellularity, DNA quality, or sequencing metrics, and we have demonstrated that the quality metrics were generally favorable and resulted in the inclusion of a greater proportion of late-stage tumors that would otherwise have been excluded. We then examined a small cohort of patients with samples collected pre- and post-chemotherapy (*n* = 10) and observed a range in the degree of genomic concordance given the extent of heterogeneity expected in this disease (Dulak et al. 2013; Nones et al. 2014; Weaver et al. 2014). For the samples within a case that showed a high degree of similarity (e.g., IDs 002 and 001), we can trace their common ancestry to a point prior to the chemotherapy. On the other hand, when the pre- and post-chemotherapy samples show substantial differences (e.g., IDs 007, 008, and 005), the timing of the divergence of the samples can be traced to being near-synchronous with endoreduplication. When investigating the effect of chemotherapy on a larger scale in a cohort of 120 patients, we observe that the genome of EAC is remarkably resistant to the effect of neoadjuvant chemotherapy. Indeed, there was a striking similarity noted between chemotherapy-naive samples and those treated with neoadjuvant chemotherapy at the level of copy-number aberrations, SNVs, and mutational spectra. This study was not designed to examine the genetic predictors of response to chemotherapy, which requires a different experimental approach given that it is generally a chemoresistant disease. Only 20% of patients in our study showed a histopathological response (Mandard score 1–3, based on the degree of fibrosis and proportion of tumor cells remaining) to chemotherapy, which is consistent with the treatment response expected from the literature (Cunningham et al. 2006; Alderson et al. 2015).

To date, most large-scale sequencing efforts, including The Cancer Genome Atlas (TCGA) and other ICGC projects, have been confined to patients who are naive to systemic treatment. Hence, for cancers treated with chemotherapy prior to surgical resection (e.g., cancers of the stomach, esophagus, breast, bladder, cervix, and lung), this has restricted the samples available for analysis to pretreatment diagnostic biopsies that are generally obtained via endoscopy or laparoscopy and are challenging to work with due to their small size. The main reason for exclusion of samples in our cohort was low cellularity (<70%) as determined by expert pathology review (three independent pathologists) of a frozen section taken from the samples used for DNA extraction. The proportion of chemotherapy-treated samples excluded at this stage was more than twice as high as the proportion of treatment-naive samples, and so this will potentially bias selection away from those who show a good histopathological response to systemic neoadjuvant treatment. However, apart from cellularity there was no further difference in the quality or quantity of DNA, library or sequence obtained. In the future as technology improves, sequencing of single cells in cases that are highly responsive to neoadjuvant therapy may be informative.

Our observation that the majority of EAC genomes remained rather stable following chemotherapy is consistent with breast cancer studies when considering those patients with chemoresistant disease. For example, in a candidate gene study of 47 breast cancer patients, Almendro et al. (2014) found that intratumor genetic diversity was indicative of the tumor subtype and remained stable in patients with only partial or no response to treatment. Yates et al. (2015) interrogated the subclonal architecture of breast cancer in 50 patients, of which 18 had samples taken before and after neoadjuvant chemotherapy. In five of these patients, new clones were seen in the post-chemotherapy samples with potential driver events such as amplifications in *MYC* and *FGFR2* and deletions in *RUNX1*. Detailed phylogenetic reconstruction of these five cases suggested that the treatment-resistant clones they observed were likely to have been missed at the time of prechemotherapy sampling, and were unlikely to be the result of new subclones arising during treatment.

In the context of EAC, Murugaesu et al. (2015) performed exome sequencing on samples from eight cases taken before and after chemotherapy. The extensive multiregion sampling was a strength of this small study, and they found a positive correlation between the degree of intra-tumoral heterogeneity and a poor response to neoadjuvant chemotherapy, which in turn correlated with a worse survival. Our study was performed as part of the ICGC, which is designed to examine the landscape by virtue of examining a large number of tumor:normal pairs, and hence, we were generally unable to perform multiregional sampling. Findlay et al. (2016) recently reported results from their exome anlaysis of 30 pre- and post-chemotherapy EAC samples, and in this study, they purposefully selected cases showing a range of responses to chemotherapy. They associated good clinical response, as determined by the histopathological Mandard score generated from the post-chemotherapy surgical resection specimen, with evidence for genomic bottlenecking as a result of chemotherapy. This is at odds with our interpretation.

We cannot, from such a limited number of cases with pre- and post-chemotherapy samples, in such a diverse disease, separate the potential sources of heterogeneity arising from spatial sampling, temporal sampling, and chemotherapy unless we can make some inference about the timing of events. It has been reported previously that some point mutations, LOHs, and genome duplication events occur early in the cancer progression and that genomic catastrophes and the accumulation of clonal diversity may play a role. Our paired cases support these prior observations (Nones et al. 2014).

On average, we noted that approximately a quarter of the genome had undergone LOH both pre- and post-chemotherapy in our samples, and in all cases, with paired samples the same allele was lost pre- and post-chemotherapy. Therefore, we infer that LOH and then genome doubling occur early in the life history of the cancers. The high point mutation rate associated with EAC allows us to say something about the timing of genomic catastrophes and the establishment of clonal diversity. If large-scale genomic rearrangements predate clonal diversity, then we expect to see SNVs that occur after the copy-number changes but that are shared pre- and-post chemotherapy. If the clonal diversity occurs before the copy-number changes, then we would expect to see SNVs that are unique to one sample but that predate local copy-number changes. We see neither of these, strongly suggesting that the establishment of clonal diversity and the copy-number changes are roughly concurrent. This suggests that it is not just localized catastrophes but genome-wide changes that seem to occur near-simultaneously. Therefore, the divergence of the clones observed pre- and post-chemotherapy must have occurred substantially before treatment was administered, and thus, chemotherapy cannot be responsible for the divergence. An alternative explantion would be selective pressures for one clone out of those available, but the larger cohorts discussed above revealed little evidence of systematic selection of this kind.

Regarding the mutational signature analysis, we used the methods of Alexandrov et al. (2013), which identified six main SNV signatures in our data, five of which have been previously described in EAC data sets (Dulak et al. 2013; Nones et al. 2014). When comparing samples taken pre- and post-chemotherapy, we observe that the signature patterns are often different between those occurring before the copy number changes and those timed as occurring after, but the more recent signature is consistent between both the pre- and post-chemotherapy samples. Thus, any apparent differences in the mutational signatures pre- and post-chemotherapy are likely attributable to cellularity-induced differences in the power to detect the recent SNVs that, by definition, have lower allele fractions.

While our study was not designed to determine the prognostic value of genomic response to chemotherapy, we acknowledge that some of the samples for which pre- and post-chemotherapy profiles differ the most (e.g., 007 and 008) are some of those with the best survival. However, we also note they are two of the cases with the best pathological TNM staging. Any approach to prognosticate based on genomic factors (e.g., perhaps following the results of Findlay et al. 2016) should at most temper established prognostic factors such as these fundamental phenotypic characteristics. Moreover, as discussed, some mutations were found to be more recurrent following chemotherapy, and this is an area ripe for further research as the appropriate cohorts become available.

In conclusion, the overall genomic profile of EAC remains similar before and after chemotherapy. The poor survival in EAC would support our findings that this cancer is resistant to chemotherapy with remarkable consistency in the genome of the primary tumor over time. Based on our findings, we would suggest that inclusion of neoadjuvant treated samples for large-scale sequencing efforts should be considered by the sequencing community. Such an approach will avoid biasing cohorts toward the earlier stages of the disease and increase the number of samples available for analysis particularly in tumor types with neoadjuvant therapy regimens. With the increasing recognition of the extent of epithelial tumor heterogeneity, large-scale efforts are essential to maximize the power of uncovering the full spectrum of mechanisms driving tumorigenesis.

## Methods

### Sample collection and processing

EAC patients were recruited prospectively from 11 sites across the UK as part of the OCCAMS (Oesophageal Clinical and Molecular Stratification) Consortium. Patients on a palliative treatment pathway, as well as those treated with radiotherapy, were excluded. The study was approved by the institutional ethics committees (REC Ns 07/H0305/52, 10/H0305/1), and all patients gave written informed consent.

Samples were obtained during either the diagnostic esophagogastroduodenoscopy or endoscpic ultrasound procedure used for staging and/or from the surgical resection specimen (Fig. 1). For each patient, blood or normal squamous esophageal samples, at least 5 cm distant from the tumor, were used as a germline reference. In 10 cases, tumor samples were taken from multiple spatially distinct sites at surgery and, in two cases, also at EGD.

All tissue samples were snap-frozen in liquid nitrogen immediately after collection and stored at −80°C. H&E-stained sections from cancer samples were reviewed independently by two expert hisptopathologists, and DNA was extracted and sequenced if tumor cellularity was ≥70%. DNA was extracted from frozen esophageal tissue using the AllPrep DNA/RNA mini kit (Qiagen) and from blood samples using the QIAamp DNA blood maxi kit (Qiagen) according to manufacturer's instructions.

### Whole-genome sequencing

As part of the ICGC, 100- to 125-bp paired-end sequencing was performed under contract by Illumina to a typical depth of at least 50×, with 94% of the known genome being sequenced to at least 8× coverage while achieving a PHRED quality of at least 30 for at least 80% of mapping bases. QC metrics were computed on a per lane basis using FastQC (http://www.bioinformatics.babraham.ac.uk/projects/fastqc) and in-house tools, enabling the identification of sequence reads that required trimming. Technical details of the sequencing metrics are given in Supplemental Table S2.

### Mutation calling

Sequence reads were aligned to the human reference genome (GRCh37 from Ensembl release 71) (Yates et al. 2016) using BWA 0.5.9 (Li and Durbin 2009). Aligned reads were then sorted into genome coordinate order and duplicate reads marked using Picard 1.115 (FixMateInformation and MarkDuplicates tools, respectively; http://broadinstitute.github.io/picard). Somatic SNVs and indels were detected using Strelka 1.0.13 (Saunders et al. 2012). To increase accuracy, additional filters were applied to high-confidence calls (those passing Stelka's filters); details are given in Supplemental Table S6. Functional annotation of the resulting variants was performed using Variant Effect Predictor (VEP release 75) (McLaren et al. 2016).

### Copy-number calling

For the large cohort, absolute copy-number alterations, cellularities, and ploidies for each sample were estimated using ASCAT-NGS v.2.1 using read counts at germline heterozygous positions estimated by GATK 3.2-2 (Van Loo et al. 2010; Nik-Zainal et al. 2012). Segments were considered amplified if the ratio of absolute copy number to ploidy exceeded two and deleted if the ratio was less than 0.5. LOH regions were defined as regions in the genome where the minor copy number was zero.

### Mutational signature analysis

Mutational signatures were identified using the methodology described by Alexandrov et al. (2013). Before running the software, common variants in the 1000 Genomes database (The 1000 Genomes Project Consortium 2015) appearing in at least 0.5% of the population were removed. The optimal number of signatures in the data set was chosen to balance the signature stability against the Frobenius reconstruction error. The cisplatin signature enrichment analysis was performed as described by Murugaesu et al. (2015).

### Multiple sample analysis

A GATK walker was used to identify a set of germline-heterozygous loci for each trio. The search was restricted to the autosomes, sites with no more than 20 germline reads were filtered by GATK (McKenna et al. 2010), sites with germline coverage between 16 and 90 with at least four copies of each allele present, sites where the strand bias lies between 0.1 and 0.9, and sites that are not in obvious regions of germline copy-number variation, identified with fastseg (Klambauer et al. 2012). This results in approximately 2 million such loci per trio. The depths of coverage and allele fractions for these loci were recorded for all samples in the trio.

To aid segmentation, a running median was applied to the depth and allele fraction data. A single segmentation of these values was created for each patient by combining, for each tumor sample, a sliding analysis-of-variance procedure and careful manual review of the genome. We erred on the side of oversegmentation as there is little to no penalty for this in the analyses that follow. The cellularity and baseline copy number for each sample was identified using the Crambled tool (Lynch 2015), and depth and allele-fraction values for clonal copy-number states were predicted. Segments were assigned to these copy-number states, or subclonal combinations of those states, based on the mean values for the segments. Where solutions for a segment appeared to be subclonal or differed between the multiple samples for a patient, they were reviewed for possible technical explanations such as missegmentation. Neighboring “segments” assigned the same copy-number state in both samples were merged. Segments were compared across samples to confirm the consistency of allele assignment (e.g., if both samples show two copies of one allele and one copy of the other, is the same allele duplicated in both cases) and corrected if not.

SNVs were called with Strelka and annotated with VEP as described elsewhere. SNVs were mapped to a copy-number state pre- and post-chemotherapy. SNVs with the same copy-number combination pre- and post-chemotherapy were partitioned into early (coming before a copy-number change) and late mutations where copy-number states and power allowed. Vectors of trinucleotide mutation counts were deconstructed into the 30 COSMIC signatures (http://cancer.sanger.ac.uk/cosmic/signatures) using a quadratic programming approach (Lynch et al. 2016).

## Data access

The whole-genome sequencing data from this study have been submitted to the European Genome-phenome Archive (EGA; https://www.ebi.ac.uk/ega/home) under accession number EGAD00001002241. Mutation calls can be found within the ICGC data portal (https://dcc.icgc.org/) under project ID ESAD-UK and library IDs listed in Supplemental Table S2.

## Members of OCCAMS Consortium

Rachael Fels Elliott,^7^ Paul A.W. Edwards,^7^ Xiaodun Li,^7^ Hamza Chettouh,^7^ Gianmarco Contini,^7^ Eleanor Gregson,^7^ Sebastian Zeki,^7^ Laura Smith,^7^ Zarah Abdullahi,^7^ Rachel de la Rue,^7^ Ahmad Miremadi,^7^,^8^ Shalini Malhotra,^7^,^8^ Mike L. Smith,^9^ Jim Davies,^10^ Charles Crichton,^11^ Nick Carroll,^12^ Peter Safranek,^12^ Andrew Hindmarsh,^12^ Vijayendran Sujendran,^12^ Richard Turkington,^13^ Stephen J. Hayes,^14^,^15^ Yeng Ang,^14^,^16^,^17^ Shaun R. Preston,^18^ Sarah Oakes,^18^ Izhar Bagwan,^18^ Vicki Save,^19^ Richard J.E. Skipworth,^19^ Ted R. Hupp,^19^ J. Robert O'Neill,^19^,^20^ Olga Tucker,^21^,^22^ Andrew Beggs,^21^,^22^ Philippe Taniere,^21^ Sonia Puig,^21^ Timothy J. Underwood,^23^,^24^ Fergus Noble,^23^ Jack Owsley,^23^ Hugh Barr,^25^ Neil Shepherd,^25^ Oliver Old,^25^ Jesper Lagergren,^26^,^27^ James Gossage,^26^,^28^ Andrew Davies,^26^,^28^ Fuju Chang,^26^,^28^ Janine Zylstra,^26^,^28^ Grant Sanders,^29^ Richard Berrisford,^29^ Catherine Harden,^29^ David Bunting,^29^ Mike Lewis,^30^ Ed Cheong,^30^ Bhaskar Kumar,^30^ Simon L. Parsons,^31^ John Saunders,^31^ Irshad Soomro,^31^ Philip Kaye,^31^ Laurence Lovat,^32^ Rehan Haidry,^32^ Victor Eneh,^32^ Laszlo Igali,^33^ Ian M. Welch,^34^ Michael Scott,^34^ Shamila Sothi,^35^ Sari Suortamo,^35^ Suzy Lishman,^36^ Anna Grabowska,^37^ Christopher J. Peters,^38^ George B. Hanna,^38^ David Khoo,^39^ Duncan Beardsmore,^40^

## Supplementary Material

Supplemental Material

## Footnotes

7 Medical Research Council Cancer Unit, Hutchison/Medical Research Council Research Centre, University of Cambridge, Cambridge CB2 0XZ, United Kingdom

8 Department of Histopathology, Addenbrooke's Hospital, Cambridge CB2 0QQ, United Kingdom

9 Cancer Research UK Cambridge Institute, University of Cambridge, Cambridge CB2 0RE, United Kingdom

10 Oxford ComLab, University of Oxford, OX1 2JD, United Kingdom

11 Department of Computer Science, University of Oxford, OX1 3QD, United Kingdom

12 Cambridge University Hospitals NHS Foundation Trust, Cambridge, CB2 0QQ, United Kingdom

13 Centre for Cancer Research and Cell Biology, Queen's University Belfast, BT9 7AB, Northern Ireland, United Kingdom

14 Salford Royal NHS Foundation Trust, Salford, M6 8HD, United Kingdom

15 Faculty of Medical and Human Sciences, University of Manchester, M13 9PL, United Kingdom

16 Wigan and Leigh NHS Foundation Trust, Wigan, Manchester, WN1 2NN, United Kingdom

17 GI Science Centre, University of Manchester, M13 9PL, United Kingdom

18 Royal Surrey County Hospital NHS Foundation Trust, Guildford, GU2 7XX, United Kingdom

19 Edinburgh Royal Infirmary, Edinburgh, EH16 4SA, United Kingdom

20 Edinburgh University, Edinburgh, EH8 9YL, United Kingdom

21 University Hospitals Birmingham NHS Foundation Trust, Birmingham, B15 2GW, United Kingdom

22 Institute of Cancer and Genomic Sciences, University of Birmingham, B15 2TT, United Kingdom

23 University Hospital Southampton NHS Foundation Trust, Southampton, SO16 6YD, United Kingdom

24 Cancer Sciences Division, University of Southampton, Southampton, SO17 1BJ, United Kingdom

25 Gloucester Royal Hospital, Gloucester, GL1 3NN, United Kingdom

26 St Thomas's Hospital, London, SE1 7EH, United Kingdom

27 Karolinska Institutet, SE-171 77, Stockholm, Sweden

28 King's College London, London, WC2R 2LS, United Kingdom

29 Plymouth Hospitals NHS Trust, Plymouth, PL6 8DH, United Kingdom

30 Norfolk and Norwich University Hospital NHS Foundation Trust, Norwich, NR4 7UY, United Kingdom

31 Nottingham University Hospitals NHS Trust, Nottingham, NG7 2UH, United Kingdom

32 University College London, London, WC1E 6BT, United Kingdom

33 Norfolk and Waveney Cellular Pathology Network, Norwich, NR4 7UY, United Kingdom

34 Wythenshawe Hospital, Manchester, M23 9LT, United Kingdom

35 University Hospitals Coventry and Warwickshire NHS Trust, Coventry, CV2 2DX, United Kingdom

36 Peterborough Hospitals NHS Trust, Peterborough City Hospital, Peterborough, PE3 9GZ, United Kingdom

37 Queen's Medical Centre, University of Nottingham, Nottingham, NG7 2UH, United Kingdom

38 Imperial College NHS Trust, Imperial College London, W2 1NY, United Kingdom

39 Barking Havering and Redbridge University Hospitals NHS Trust, Queen's Hospital, Romford RM7 0AG, United Kingdom

40 Royal Stoke University Hospital, Stoke-on-Trent, ST4 6QG, United Kingdom

## References

[NOORANIGR214296C1] The 1000 Genomes Project Consortium. 2015 A global reference for human genetic variation. Nature 526: 68–74. 26432245 10.1038/nature15393 PMC4750478

[NOORANIGR214296C2] Alderson D, Langley RE, Nankivell MG, Blazeby JM, Griffin M, Crellin A, Grabsch HI, Okines AFC, Goldstein C, Falk S, 2015 Neoadjuvant chemotherapy for resectable oesophageal and junctional adenocarcinoma: results from the UK Medical Research Council randomised OEO5 trial (ISRCTN 01852072). J Clin Oncol 33: abstract 4002.

[NOORANIGR214296C3] Alexandrov LB, Nik-Zainal S, Wedge DC, Aparicio SA, Behjati S, Biankin AV, Bignell GR, Bolli N, Borg A, Borresen-Dale AL, 2013 Signatures of mutational processes in human cancer. Nature 500: 415–421. 23945592 10.1038/nature12477 PMC3776390

[NOORANIGR214296C4] Allum WH, Blazeby JM, Griffin SM, Cunningham D, Jankowski JA, Wong R; Association of Upper Gastrointestinal Surgeons of Great Britain and Ireland, the British Soceity of Gastroenterologyand the British Association of Surgical Oncology. 2011 Guidelines for the management of oesophageal and gastric cancer. Gut 60: 1449–1472. 21705456 10.1136/gut.2010.228254

[NOORANIGR214296C5] Almendro V, Cheng YK, Randles A, Itzkovitz S, Marusyk A, Ametller E, Gonzalez-Farre X, Munoz M, Russnes HG, Helland A, 2014 Inference of tumor evolution during chemotherapy by computational modeling and in situ analysis of genetic and phenotypic cellular diversity. Cell Rep 6: 514–527. 24462293 10.1016/j.celrep.2013.12.041 PMC3928845

[NOORANIGR214296C6] Cunningham D, Allum WH, Stenning SP, Thompson JN, Van de Velde CJ, Nicolson M, Scarffe JH, Lofts FJ, Falk SJ, Iveson TJ, 2006 Perioperative chemotherapy versus surgery alone for resectable gastroesophageal cancer. N Engl J Med 355: 11–20. 16822992 10.1056/NEJMoa055531

[NOORANIGR214296C7] Dulak AM, Stojanov P, Peng S, Lawrence MS, Fox C, Stewart C, Bandla S, Imamura Y, Schumacher SE, Shefler E, 2013 Exome and whole-genome sequencing of esophageal adenocarcinoma identifies recurrent driver events and mutational complexity. Nat Genet 45: 478–486. 23525077 10.1038/ng.2591 PMC3678719

[NOORANIGR214296C8] Findlay JM, Castro-Giner F, Makino S, Rayner E, Kartsonaki C, Cross W, Kovac M, Ulahannan D, Palles C, Gillies RS, 2016 Differential clonal evolution in oesophageal cancers in response to neo-adjuvant chemotherapy. Nat Commun 7: 11111. 27045317 10.1038/ncomms11111 PMC4822033

[NOORANIGR214296C9] Jemal A, Bray F, Center MM, Ferlay J, Ward E, Forman D. 2011 Global cancer statistics. CA Cancer J Clin 61: 69–90. 21296855 10.3322/caac.20107

[NOORANIGR214296C10] Klambauer G, Schwarzbauer K, Mayr A, Clevert DA, Mitterecker A, Bodenhofer U, Hochreiter S. 2012 cn.MOPS: mixture of Poissons for discovering copy number variations in next-generation sequencing data with a low false discovery rate. Nucleic Acids Res 40: e69. 22302147 10.1093/nar/gks003 PMC3351174

[NOORANIGR214296C11] Lepage C, Drouillard A, Jouve JL, Faivre J. 2013 Epidemiology and risk factors for oesophageal adenocarcinoma. Dig Liver Dis 45: 625–629. 23453359 10.1016/j.dld.2012.12.020

[NOORANIGR214296C12] Li H, Durbin R. 2009 Fast and accurate short read alignment with Burrows-Wheeler transform. Bioinformatics 25: 1754–1760. 19451168 10.1093/bioinformatics/btp324 PMC2705234

[NOORANIGR214296C13] Lynch A. 2015 Crambled: a Shiny application to enable intuitive resolution of conflicting cellularity estimates. F1000Res 4: 1407. 26962434 10.12688/f1000research.7453.1 PMC4765721

[NOORANIGR214296C14] Lynch MJ, Mulvaney MJ, Hodges SC, Thompson TL, Thomason WE. 2016 Decomposition, nitrogen and carbon mineralization from food and cover crop residues in the central plateau of Haiti. Springerplus 5: 973. 27429883 10.1186/s40064-016-2651-1 PMC4932013

[NOORANIGR214296C15] Masclee GM, Coloma PM, de Wilde M, Kuipers EJ, Sturkenboom MC. 2014 The incidence of Barrett's oesophagus and oesophageal adenocarcinoma in the United Kingdom and The Netherlands is levelling off. Aliment Pharmacol Ther 39: 1321–1330. 24738722 10.1111/apt.12759

[NOORANIGR214296C16] McKenna A, Hanna M, Banks E, Sivachenko A, Cibulskis K, Kernytsky A, Garimella K, Altshuler D, Gabriel S, Daly M, 2010 The Genome Analysis Toolkit: a MapReduce framework for analyzing next-generation DNA sequencing data. Genome Res 20: 1297–1303. 20644199 10.1101/gr.107524.110 PMC2928508

[NOORANIGR214296C17] McLaren W, Gil L, Hunt SE, Riat HS, Ritchie GR, Thormann A, Flicek P, Cunningham F. 2016 The Ensembl Variant Effect Predictor. Genome Biol 17: 122. 27268795 10.1186/s13059-016-0974-4 PMC4893825

[NOORANIGR214296C18] Medical Research Council Oesophageal Cancer Working Group. 2002 Surgical resection with or without preoperative chemotherapy in oesophageal cancer: a randomised controlled trial. Lancet 359: 1727–1733. 12049861 10.1016/S0140-6736(02)08651-8

[NOORANIGR214296C19] Meier B, Cooke SL, Weiss J, Bailly AP, Alexandrov LB, Marshall J, Raine K, Maddison M, Anderson E, Stratton MR, 2014 *C. elegans* whole-genome sequencing reveals mutational signatures related to carcinogens and DNA repair deficiency. Genome Res 24: 1624–1636. 25030888 10.1101/gr.175547.114 PMC4199376

[NOORANIGR214296C20] Murugaesu N, Wilson GA, Birkbak NJ, Watkins TB, McGranahan N, Kumar S, Abbassi-Ghadi N, Salm M, Mitter R, Horswell S, 2015 Tracking the genomic evolution of esophageal adenocarcinoma through neoadjuvant chemotherapy. Cancer Discov 5: 821–831. 26003801 10.1158/2159-8290.CD-15-0412 PMC4529488

[NOORANIGR214296C21] Nik-Zainal S, Van Loo P, Wedge DC, Alexandrov LB, Greenman CD, Lau KW, Raine K, Jones D, Marshall J, Ramakrishna M, 2012 The life history of 21 breast cancers. Cell 149: 994–1007. 22608083 10.1016/j.cell.2012.04.023 PMC3428864

[NOORANIGR214296C22] Nones K, Waddell N, Wayte N, Patch AM, Bailey P, Newell F, Holmes O, Fink JL, Quinn MC, Tang YH, 2014 Genomic catastrophes frequently arise in esophageal adenocarcinoma and drive tumorigenesis. Nat Commun 5: 5224. 25351503 10.1038/ncomms6224 PMC4596003

[NOORANIGR214296C23] Orditura M, Galizia G, Di Martino N, Ancona E, Castoro C, Pacelli R, Morgillo F, Rossetti S, Gambardella V, Farella A, 2014 Effect of preoperative chemoradiotherapy on outcome of patients with locally advanced esophagogastric junction adenocarcinoma—a pilot study. Current Oncol 21: 125–133. 10.3747/co.21.1570 PMC4059797 24940093

[NOORANIGR214296C24] Rebucci M, Michiels C. 2013 Molecular aspects of cancer cell resistance to chemotherapy. Biochem Pharmacol 85: 1219–1226. 23435357 10.1016/j.bcp.2013.02.017

[NOORANIGR214296C25] Saunders CT, Wong WS, Swamy S, Becq J, Murray LJ, Cheetham RK. 2012 Strelka: accurate somatic small-variant calling from sequenced tumor-normal sample pairs. Bioinformatics 28: 1811–1817. 22581179 10.1093/bioinformatics/bts271

[NOORANIGR214296C26] Sjoquist KM, Burmeister BH, Smithers BM, Zalcberg JR, Simes RJ, Barbour A, Gebski V, Australasian Gastro-Intestinal Trials Group. 2011 Survival after neoadjuvant chemotherapy or chemoradiotherapy for resectable oesophageal carcinoma: an updated meta-analysis. Lancet Oncol 12: 681–692. 21684205 10.1016/S1470-2045(11)70142-5

[NOORANIGR214296C27] Van Loo P, Nordgard SH, Lingjaerde OC, Russnes HG, Rye IH, Sun W, Weigman VJ, Marynen P, Zetterberg A, Naume B, 2010 Allele-specific copy number analysis of tumors. Proc Natl Acad Sci 107: 16910–16915. 20837533 10.1073/pnas.1009843107 PMC2947907

[NOORANIGR214296C28] Weaver JM, Ross-Innes CS, Shannon N, Lynch AG, Forshew T, Barbera M, Murtaza M, Ong CA, Lao-Sirieix P, Dunning MJ, 2014 Ordering of mutations in preinvasive disease stages of esophageal carcinogenesis. Nat Genet 46: 837–843. 24952744 10.1038/ng.3013 PMC4116294

[NOORANIGR214296C29] Woods D, Turchi JJ. 2013 Chemotherapy induced DNA damage response: convergence of drugs and pathways. Cancer Biol Ther 14: 379–389. 23380594 10.4161/cbt.23761 PMC3672181

[NOORANIGR214296C30] Yates LR, Gerstung M, Knappskog S, Desmedt C, Gundem G, Van Loo P, Aas T, Alexandrov LB, Larsimont D, Davies H, 2015 Subclonal diversification of primary breast cancer revealed by multiregion sequencing. Nat Med 21: 751–759. 26099045 10.1038/nm.3886 PMC4500826

[NOORANIGR214296C31] Yates A, Akanni W, Amode MR, Barrell D, Billis K, Carvalho-Silva D, Cummins C, Clapham P, Fitzgerald S, Gil L, 2016 Ensembl 2016. Nucleic Acids Res 44: D710–D716. 26687719 10.1093/nar/gkv1157 PMC4702834

