# Supplementary material for: A comparative analysis of whole genome sequencing of esophageal adenocarcinoma pre- and post-chemotherapy
Source: Genome Res. 2017 Jun;27(6):902–12. doi: 10.1101/gr.214296.116 (PMC5453324; doi:10.1101/gr.214296.116)
Supplement: Supplemental Material [file supp_27_6_902__index.html]

A comparative analysis of whole genome sequencing of oesophageal adenocarcinoma pre- and post-chemotherapy — Supplemental Material 

# A comparative analysis of whole genome sequencing of esophageal adenocarcinoma pre- and post-chemotherapy

## Supplemental Material

- Supplemental\_Fig\_S1.docx
- Supplemental\_Fig\_S2.docx
- Supplemental\_Fig\_S3.docx
- Supplemental\_Fig\_S4.docx
- Supplemental\_Fig\_S5.docx
- Supplemental\_Fig\_S6.docx
- Supplemental\_Table\_S1.docx
- Supplemental\_Table\_S2.xlsx
- Supplemental\_Table\_S3.xlsx
- Supplemental\_Table\_S4.docx
- Supplemental\_Table\_S5.docx
- Supplemental\_Table\_S6.docx
- Supplemental\_Table\_S7.docx
- Supplemental\_Table\_\_S8.xlsx
