# Supplementary material for: A comparative analysis of whole genome sequencing of esophageal adenocarcinoma pre- and post-chemotherapy
Source: Genome Res. 2017 Jun;27(6):902–12. doi: 10.1101/gr.214296.116 (PMC5453324; doi:10.1101/gr.214296.116)
Supplement: Supplemental Material [file supp_gr.214296.116_Supplemental_Fig_S1.docx]

**Supplemental Figure 1.**

**Supplemental Figure 1. SNVs affecting key genes.** For TP53, SMAD4, ARID1A, CDKN2A, MYO18B, SEMA5A, SYNE1, DOCK2, CNTNAP5 details of the allele-specific copy number for each sample are given (number of squares, allele indicated by colour). The presence of an SNV within the gene is indicated by a filled circle.
