# Supplementary material for: A comparative analysis of whole genome sequencing of esophageal adenocarcinoma pre- and post-chemotherapy
Source: Genome Res. 2017 Jun;27(6):902–12. doi: 10.1101/gr.214296.116 (PMC5453324; doi:10.1101/gr.214296.116)
Supplement: Supplemental Material [file supp_gr.214296.116_Supplemental_Fig_S2.docx]

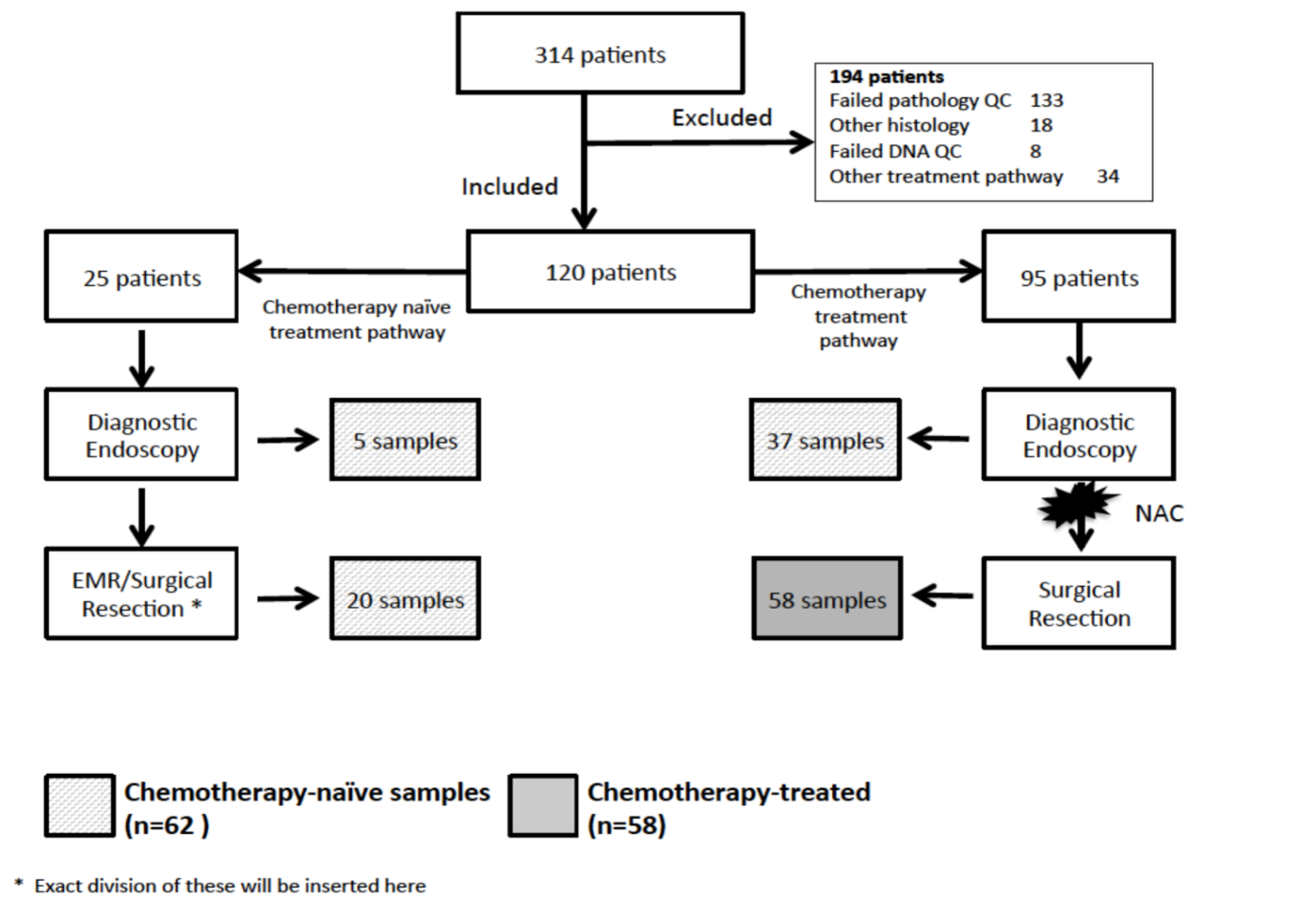


b)

a)

**
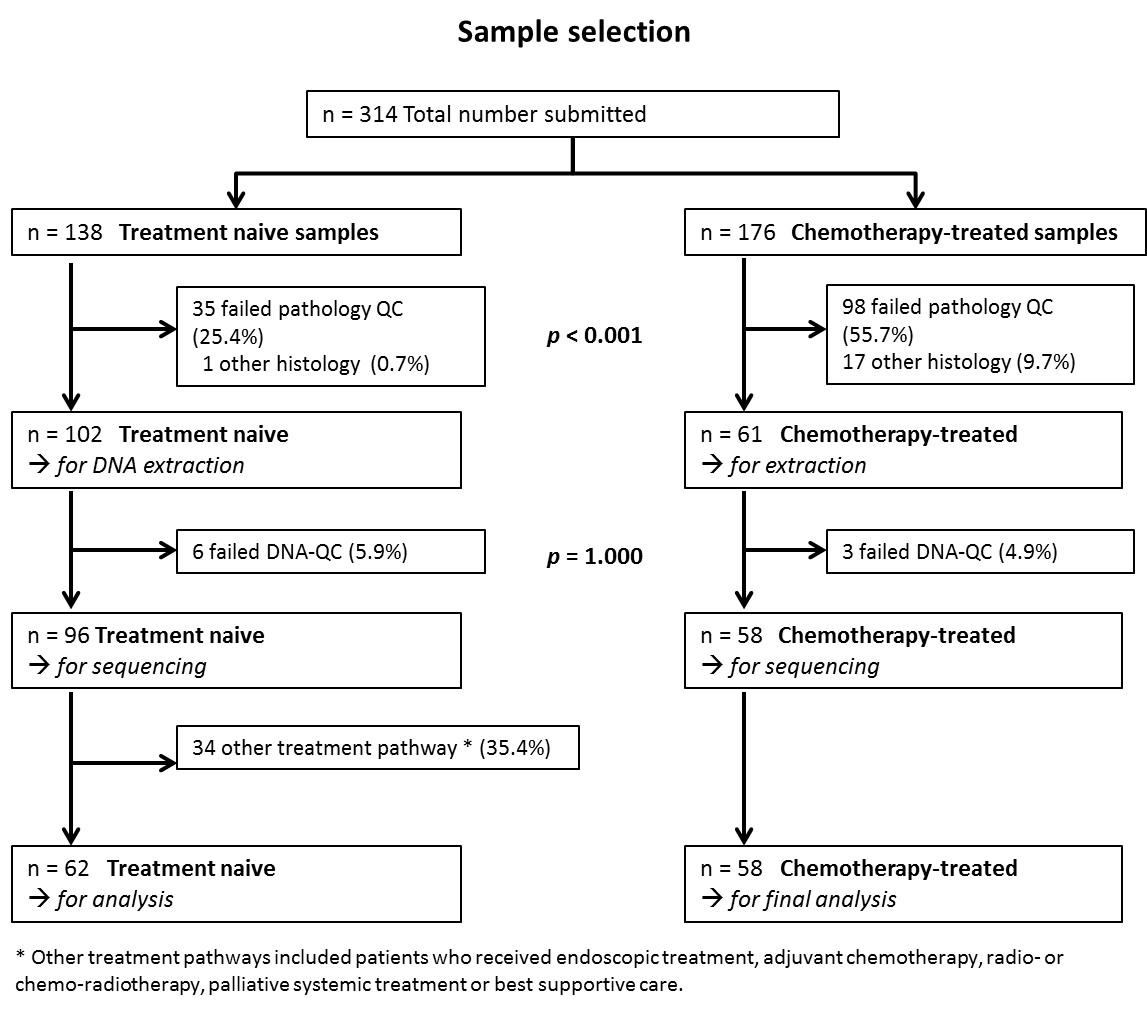
**

**Supplemental Figure 2 a). Treatment pathways and sampling of the study cohort**. Following rigorous quality control, 120 patients were included in the final cohort. Patients underwent either neoadjuvant chemotherapy followed by surgical resection or resection without any chemotherapy. Chemotherapy-treated samples were taken at surgery after neoadjuvant treatment was completed. Chemotherapy-naive samples were taken at initial endoscopy at diagnosis in patients who later went on to receive neoadjuvant treatment, or either at initial diagnosis or at resection in patients who went straight to resection without having any chemotherapy. Only one sample was included per patient. NAC= neoadjuvant chemotherapy. **b). Sample selection.** Breakdown of the pipeline showing samples selected for the final analysis of treatment naive and chemotherapy treated samples.
