# Supplementary material for: A comparative analysis of whole genome sequencing of esophageal adenocarcinoma pre- and post-chemotherapy
Source: Genome Res. 2017 Jun;27(6):902–12. doi: 10.1101/gr.214296.116 (PMC5453324; doi:10.1101/gr.214296.116)
Supplement: Supplemental Material [file supp_gr.214296.116_Supplemental_Fig_S3.docx]

##
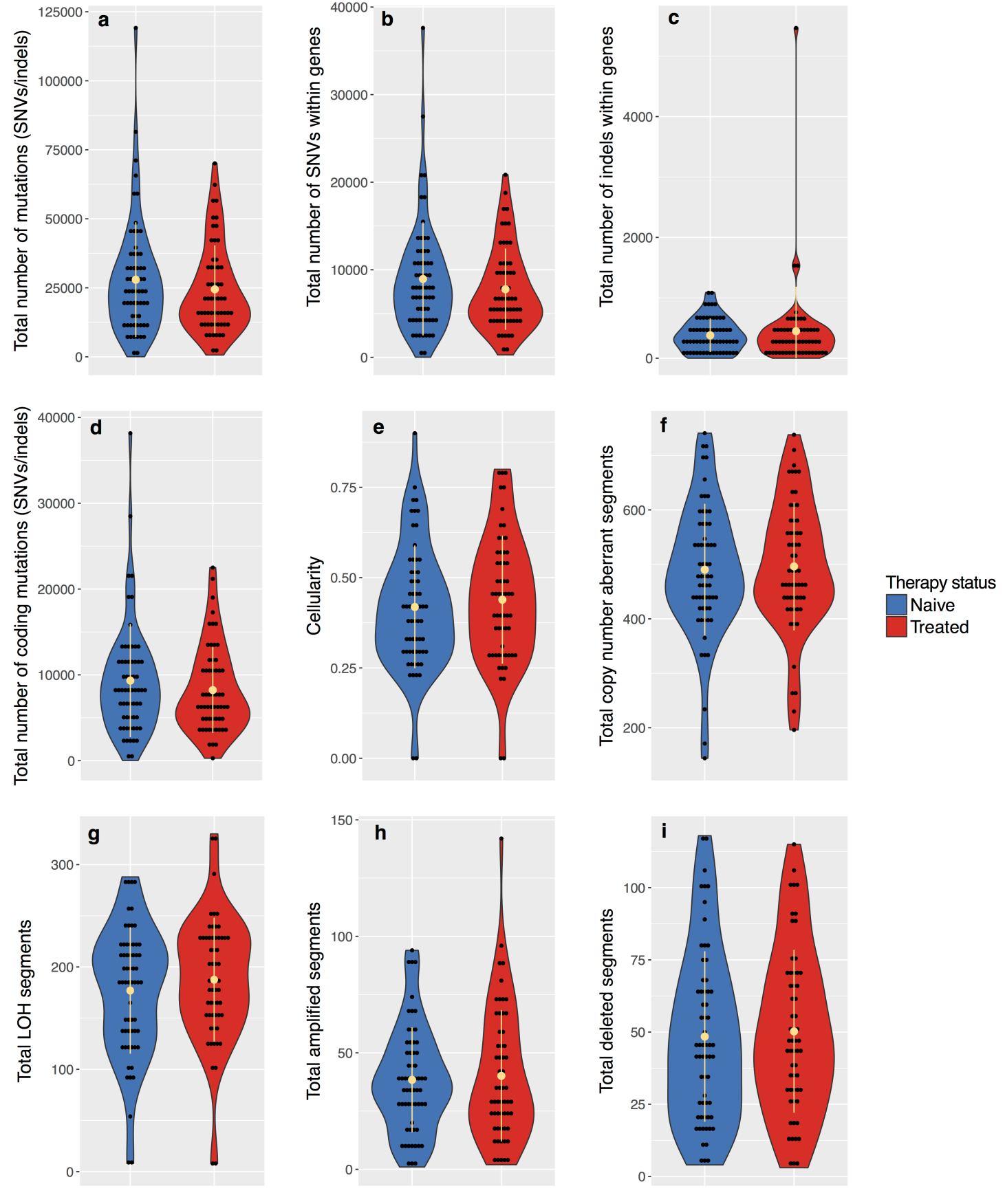


## Supplemental Figure 3. Additional genomic features of chemotherapy naive (n=62) and chemotherapy treated (n=58) samples. a. Total number of mutations (SNVs and indels), b. Total number of SNVs affecting genes, c. Total number of indels affecting genes, d. Total number of mutations falling within genes (SNVs and Indels), e. Cellularity, f. Total copy number aberrant segments, g. Total LOH segments, h. Total amplified segments, i. Total deleted segments. The mean ± 1 standard deviation are highlighted in yellow in each case.
