# Supplementary material for: A comparative analysis of whole genome sequencing of esophageal adenocarcinoma pre- and post-chemotherapy
Source: Genome Res. 2017 Jun;27(6):902–12. doi: 10.1101/gr.214296.116 (PMC5453324; doi:10.1101/gr.214296.116)
Supplement: Supplemental Material [file supp_gr.214296.116_Supplemental_Fig_S4.docx]

## Supplemental Figure 4. Mutation counts at each of the 96 trinucleotide contexts, compared between the naïve and chemotherapy treated groups. No significant difference was observed between groups for any substitution class (Wilcoxon rank-sum test).

**
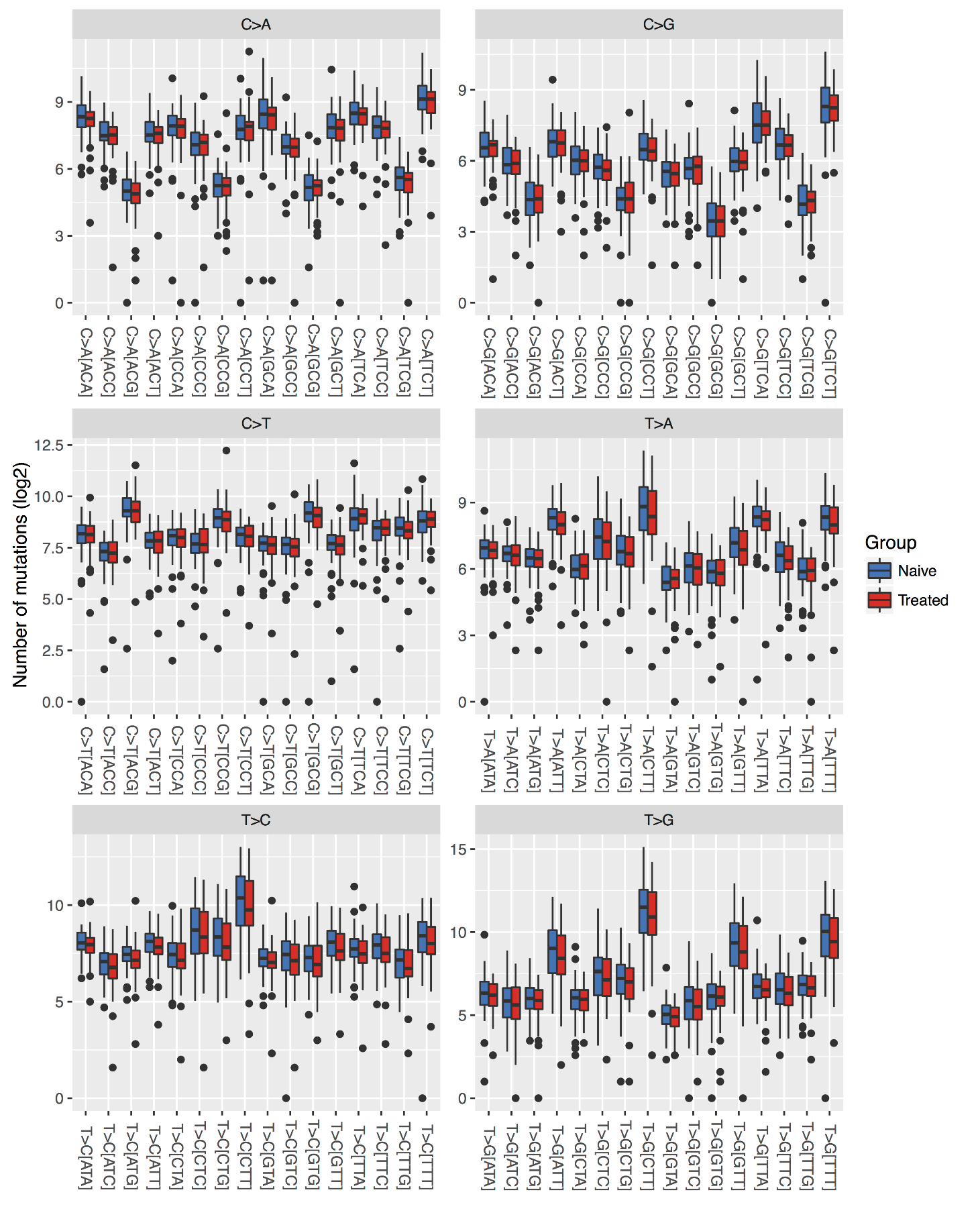
**
