# Supplementary material for: A comparative analysis of whole genome sequencing of esophageal adenocarcinoma pre- and post-chemotherapy
Source: Genome Res. 2017 Jun;27(6):902–12. doi: 10.1101/gr.214296.116 (PMC5453324; doi:10.1101/gr.214296.116)
Supplement: Supplemental Material [file supp_gr.214296.116_Supplemental_Fig_S5.docx]

##
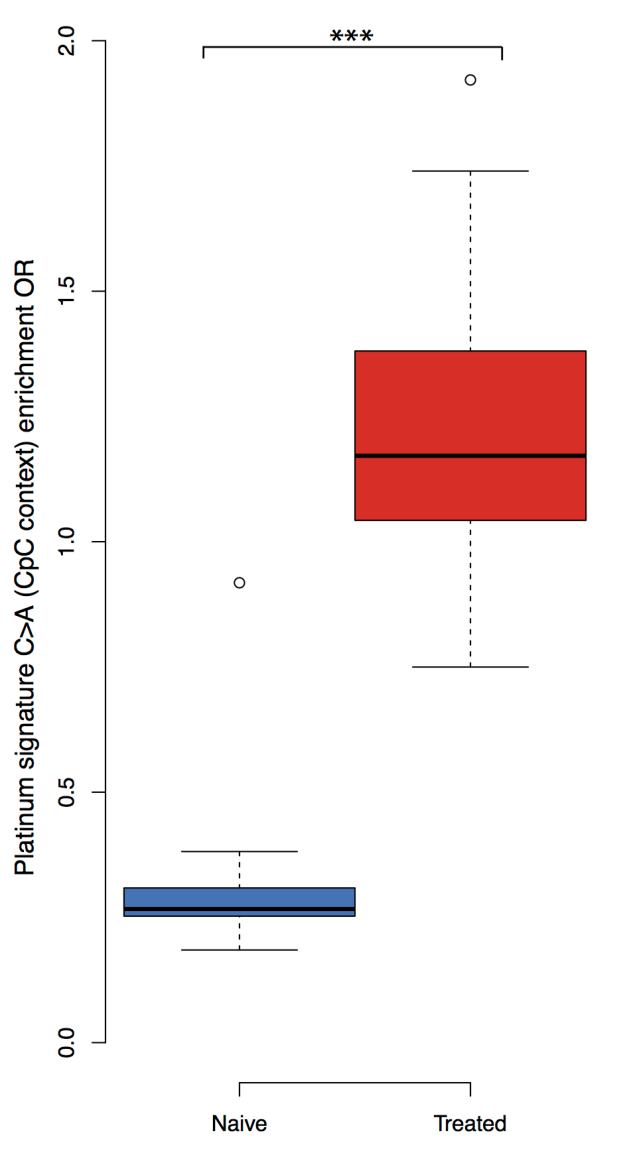


**Supplemental Figure 5. Platinum signature enrichment odds ratio for chemotherapy naive (n=62) and chemotherapy treated (n=58) samples.** The platinum signature encompasses C>A (G>T) mutations in a CpC (GpG) context. A significant enrichment of a platinum signature is observed in the chemotherapy treated group (Fisher exact test p-value<<0.0001).
