# Supplementary material for: A comparative analysis of whole genome sequencing of esophageal adenocarcinoma pre- and post-chemotherapy
Source: Genome Res. 2017 Jun;27(6):902–12. doi: 10.1101/gr.214296.116 (PMC5453324; doi:10.1101/gr.214296.116)
Supplement: Supplemental Material [file supp_gr.214296.116_Supplemental_Fig_S6.docx]

##

Signature breakdown pre-chemotherapy

##

Other

Signature 2

Signature 5

Signature 9

Signature 8

##

Signature 3

Signature 18

Signature 1

Signature 17

Signature breakdown post-chemotherapy

**Supplemental Figure 6.** **Comparison of the mutational signatures and trinucleotide contexts for the 10 pairs of chemotherapy naive and treated patients.** Mutational signatures were extracted using the methods presented in Alexandov et al 2013.
