# Supplementary material for: A comparative analysis of whole genome sequencing of esophageal adenocarcinoma pre- and post-chemotherapy
Source: Genome Res. 2017 Jun;27(6):902–12. doi: 10.1101/gr.214296.116 (PMC5453324; doi:10.1101/gr.214296.116)
Supplement: Supplemental Material [file supp_gr.214296.116_Supplemental_Table_S1.docx]

**Supplemental Table 1. Clinical demographics of the matched pre and post chemotherapy cohort.** Abbreviations: CisFU: cisplatin & 5-fluorouracil; ECX: epirubicin & cisplatin & capecitabine (= Xeloda); EOX: epirubicin & oxplatin & capecitabine (= Xeloda); M: male; F: Female; TRG: tumor regression grade according to Mandard score.

| **ID** | **001** | **002** | **003** | **004** | **005** | **006** | **007** | **008** | **010** | **011** |
| --- | --- | --- | --- | --- | --- | --- | --- | --- | --- | --- |
| **Age at Diagnosis (yrs)** | 59.8 | 75.4 | 59.3 | 73.4 | 59.3 | 75.4 | 53.2 | 64.8 | 75.3 | 58.8 |
| **Gender** | M | M | M | F | M | M | M | M | M | M |
| **Differentiation** | Poor | Poor | Moderate | Moderate | Poor | Poor | Poor | Poor | Moderate | Poor |
| **Samples at endoscopy** | 1 | 1 | 1 | 2 | 1 | 1 | 1 | 1 | 1 | 1 |
| **Samples at Surgery** | 1 | 1 | 1 | 3 | 3 | 1 | 1 | 1 | 1 | 1 |
| **Chemotherapy Regime** | CisFU | CisFU | ECX | ECX | EOX | CisFU | CisFU | ECX | CisFU | ECX |
| **Mandard Score** | TRG4 | TRG5 | TRG4 | TRG5 | TRG4 | TRG5 | TRG5 | TRG4 | TRG3 | TRG4 |
| **Pathological TNM** | T3N1 | T3N3 | T3N2 | T3N3 | T3N3 | T1N1 | T1N0 | T2N0 | T2N1 | T2N0 |
| **Radiological Response** | Stable | Stable | Stable | Stable |  | Stable | Stable | Partial | Stable | Unknown |
| **Survival (months)** | 13.2 | 39.0 | 25.2 | 7.7 | 42.4 | 52.6 | 53.5 | 54.3 | 32.0 | 34.5 |
| **Recurrence** | Brain | Liver | Nodal | Brain | Local | - | - | - | Pancreatic | Local |
| **Time to Recurrence (months)** | 8.2 | 3.2 | 11.1 | 1.7 | 24.6 | - | - | - | 9.6 | 11.8 |
| **Survival Status** | Dead | Dead | Dead | Dead | Dead | Alive | Alive | Alive | Dead | Dead |
