# Supplementary material for: A comparative analysis of whole genome sequencing of esophageal adenocarcinoma pre- and post-chemotherapy
Source: Genome Res. 2017 Jun;27(6):902–12. doi: 10.1101/gr.214296.116 (PMC5453324; doi:10.1101/gr.214296.116)
Supplement: Supplemental Material [file supp_gr.214296.116_Supplemental_Table_S4.docx]

**Supplemental Table 4.** Comparison of sequencing outcome measures between treatment-naive and chemo-treated samples and the corresponding p-values for the Wilcoxon tests.

| **Measurement** | **Median (naive)** | **Median (treated)** | **95% confidence intervals for the difference** | **Wilcoxon test**  **p-value** |
| --- | --- | --- | --- | --- |
| *Cellularity estimate* | 0.41 | 0.44 | (-0.08, 0.04) | 0.48 |
| *Tumor average ploidy* | 2.92 | 2.82 | (-0.41, 0.35) | 0.93 |
| *Total number of SNVs called* | 23096 | 19325.5 | (-2873.1,10088.9) | 0.40 |
| *Total number of indels called* | 966.5 | 885 | (-628.7, 363.8) | 0.74 |
| *Total number of SNVs/indels affecting genes* | 8197 | 6679 | (-1001.8, 3214.0) | 0.40 |
| *Total number of CNA events* | 482 | 485.5 | (-49.1, 37.1) | 0.80 |
| *Total number of LOH events* | 189.5 | 186 | (-32.7, 11.5) | 0.33 |
| *Proportion of the genome containing amplifications* | 0.01 | 0.01 | (-0.003, 0.005) | 0.83 |
| *Proportion of the genome containing deletions* | 0.04 | 0.05 | (-0.02, 0.01) | 0.57 |
| *Proportion of the genome containing LOH* | 0.30 | 0.32 | (-0.08, 0.03) | 0.37 |
