# Supplementary material for: A comparative analysis of whole genome sequencing of esophageal adenocarcinoma pre- and post-chemotherapy
Source: Genome Res. 2017 Jun;27(6):902–12. doi: 10.1101/gr.214296.116 (PMC5453324; doi:10.1101/gr.214296.116)
Supplement: Supplemental Material [file supp_gr.214296.116_Supplemental_Table_S5.docx]

**Supplemental Table 5.** Comparison of sequencing outcome measures by age, disease stage, resection margin and sample source, and the corresponding p-values for ANOVA, Wilcoxon or Fisher tests.

|  | **Age (<60, [60,75), >=75)** | | **Stage (early/late)** | | **Resection margin** | | **Sample source** | |
| --- | --- | --- | --- | --- | --- | --- | --- | --- |
| **Measurement** | ***ANOVA (p-value/adjusted p-value)*** | | ***Wilcoxon test (p-value/adjusted p-value)*** | | | | | |
| *Cellularity estimate* | 0.071 | 0.67 | 0.857 | 0.892 | 0.264 | 0.528 | 0.075 | 0.412 |
| *Tumor average ploidy* | 0.873 | 1 | 0.067 | 0.335 | 0.848 | 0.848 | 0.980 | 0.980 |
| *Total number of SNVs called* | 0.962 | 1 | 0.415 | 0.692 | 0.077 | 0.528 | 0.192 | 0.412 |
| *Total number of indels called* | 0.157 | 0.67 | 0.339 | 0.678 | 0.244 | 0.528 | 0.923 | 0.980 |
| *Total number of SNVs/indels affecting genes* | 0.353 | 0.73 | 0.101 | 0.337 | 0.226 | 0.528 | 0.866 | 0.980 |
| *Total number of CNA events* | 0.423 | 0.73 | 0.892 | 0.892 | 0.472 | 0.643 | 0.705 | 0.980 |
| *Total number of LOH events* | 0.438 | 0.73 | 0.051 | 0.335 | 0.454 | 0.643 | 0.159 | 0.412 |
| *Proportion of the genome containing amplifications* | 1 | 1 | 0.575 | 0.719 | 0.578 | 0.643 | 0.809 | 0.980 |
| *Proportion of the genome containing deletions* | 0.201 | 0.67 | 0.539 | 0.719 | 0.562 | 0.643 | 0.206 | 0.412 |
| *Proportion of the genome containing LOH* | 0.785 | 1 | 0.181 | 0.453 | 0.237 | 0.528 | 0.108 | 0.412 |
