# Supplementary material for: A comparative analysis of whole genome sequencing of esophageal adenocarcinoma pre- and post-chemotherapy
Source: Genome Res. 2017 Jun;27(6):902–12. doi: 10.1101/gr.214296.116 (PMC5453324; doi:10.1101/gr.214296.116)
Supplement: Supplemental Material [file supp_gr.214296.116_Supplemental_Table_S6.docx]

**Supplemental Table 6: Summary of filters applied to single nucleotide variant (SNV) calls from Strelka.** Filters were created and thresholds chosen by assessing kernel density plots of true and false positive SNV calls for a medulloblastoma International Cancer Genome Consortium (ICGC) benchmark dataset.

| **Filter** | **Description** |
| --- | --- |
| DistanceToAlignmentEndMedian | The median shortest distance of the variant position within the read to either aligned end is less than 10 |
| DistanceToAlignmentEndMAD | The median absolute deviation of the shortest distance of the variant position within the read to either aligned end is less than 3 |
| LowMapQual | The proportion of reads at the variant position with low mapping quality (less than 1) is greater than 10% |
| MapQualDiffMedian | The difference in the median mapping quality of variant reads (in the tumor) and reference reads (in the normal) is greater than 5 |
| VariantMapQualMedian | The median mapping quality of variant reads is less than 40 |
| VariantBaseQualMedian | The median base quality at the variant position of variant reads is less than 30 |
| VariantAlleleCount | The number of variant-supporting reads in the tumor is less than 4 |
| VariantAlleleCountControl | The number of variant-supporting reads in the normal is greater than 1 |
| StrandBias | The strand bias for variant reads covering the variant position, i.e. the fraction of reads in either direction, is less than 0.02, unless the strand bias for all reads is also less than 0.02. |
| Repeat | The length of repetitive sequence adjacent to the variant position, where repeats can be 1-, 2-, 3-, or 4-mers, is 12 or more |
| SNVCluster50 | The largest number of variant positions within any 50 base pair window surrounding, but excluding, the variant position is greater than 2; variant positions are those in which the number of alternate allele is supported by at least 2 reads and at least 5% of all reads covering that position. |
| SNVCluster100 | The largest number of variant positions within any 100 base pair window surrounding, but excluding, the variant position is greater than 4; variant positions are those in which the number of alternate allele is supported by at least 2 reads and at least 5% of all reads covering that position. |
