# Supplementary material for: A comparative analysis of whole genome sequencing of esophageal adenocarcinoma pre- and post-chemotherapy
Source: Genome Res. 2017 Jun;27(6):902–12. doi: 10.1101/gr.214296.116 (PMC5453324; doi:10.1101/gr.214296.116)
Supplement: Supplemental Material [file supp_gr.214296.116_Supplemental_Table_S7.docx]

**Supplemental Table 7: Treatment regimens and pathological response for patients having received neoadjuvant treatment (naive: n=37, treated: n=58).** Abbreviations: CF: Cisplatin+5-Fluorouracil, CX: Cisplatin+Capecitabine; # MAGIC based: Epirubicin+platinum derivative+5-Fluorouracil derivative; § Other: including antibodies (e.g. bevacizumab) or taxanes (e.g. docetaxel). Histological response according to Mandard, TRG: tissue regression grade, TRG 1-3 were regarded as responders. Percentages represent partitions of each factor within either naive or treated samples.

|  |  | **Naive** |  | **Treated** |  | **Total** |  | **missing** |
| --- | --- | --- | --- | --- | --- | --- | --- | --- |
| **Regimen** | **CF/CX** | 3 | 8.3% | 20 | 34.5% | 23 | 24.5% | *n=1* |
|  | **MAGIC based #** | 27 | 75.0% | 30 | 51.7% | 57 | 60.6% |  |
|  | **Other §** | 6 | 16.7% | 8 | 13.8% | 14 | 14.9% |  |
| **Histol. Resp.** | **Responder** | 10 | 35.7% | 6 | 12.0% | 16 | 20.5% | *n=17* |
|  | **TRG 1** | 4 | 14.3% | 0 | 0.0% | 4 | 5.1% | *n=17* |
|  | **TRG 2** | 1 | 3.6% | 0 | 0.0 % | 1 | 1.3% |  |
|  | **TRG 3** | 5 | 17.9% | 6 | 12.0% | 11 | 14.1% |  |
|  | **TRG 4** | 13 | 46.4% | 28 | 56.0% | 41 | 52.6% |  |
|  | **TRG 5** | 5 | 17.9% | 16 | 32.0% | 21 | 26.9% |  |
